# Structural and Electronic Properties of Poly(ethylene terephthalate) (PET) from Polarizable Molecular Dynamics Simulations

**DOI:** 10.1021/acs.macromol.4c02109

**Published:** 2024-11-08

**Authors:** Marcelo D. Polêto, Justin A. Lemkul

**Affiliations:** † Department of Biochemistry, Virginia Tech, Blacksburg, Virginia 24061, United States; ‡ Center for Drug Discovery, Virginia Tech, Blacksburg, Virginia 24061, United States

## Abstract

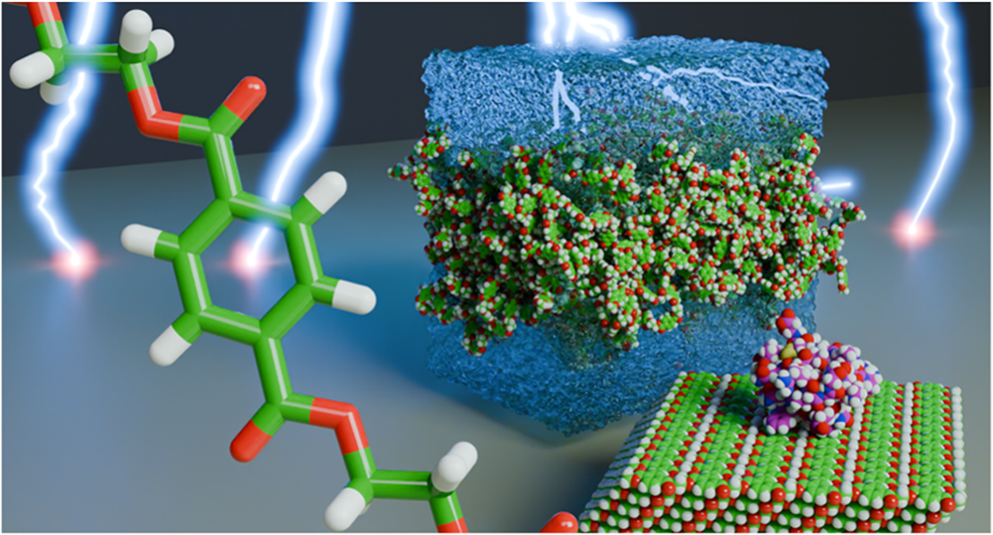

The environmental and economic challenges posed by the widespread use and disposal of plastics, particularly poly(ethylene terephthalate) (PET), require innovative solutions to mitigate their impact. Such mitigation begins with understanding physical properties of the polymer that could enable new recycling technologies. Although molecular simulations have provided valuable insights into PET interactions with various PET hydrolases, current nonpolarizable force fields neglect the electronic polarization effects inherent to PET interactions. Here, we present parameters for PET polymer and its derivatives that are compatible with the Drude polarizable force field. Our parameter fitting protocol accurately reproduces electrostatic properties from quantum mechanical calculations. We then studied electronic properties of PET amorphous slabs and PET crystal units, revealing a crucial electronic polarization response of PET residues at the interface with water or vacuum, yielding insights into the modulation of electrostatic properties by solvent molecules. Finally, we showcase the interaction between a carbohydrate-binding protein and the PET crystal unit, revealing the role of electronic polarization in enhancing binding affinity. This study represents the first extension of the Drude polarizable force field to a synthetic polymer, offering a robust tool for exploring PET material properties and advancing the design of efficient (bio)technologies for addressing plastic pollution.

## Introduction

Plastics are good examples of the “take-make-waste” production model, with the vast majority of its production coming from fossil petrochemical feedstocks and most of its disposal relying on landfilling or incineration.^1^ These practices lead to severe, negative environmental and economic impacts due to their accumulation and biodegradation recalcitrance.^1−3^ Due to the nonrenewable origin of plastics, such a paradigm also raises questions about its scarcity and potential impact on production chains worldwide in the near future.^4^

Poly(ethylene terephthalate) (PET) is one of the most single-used plastics produced worldwide,^5,6^ with wide applicability in the textile fiber and packing industries.^7^ The most common recycling process applied to PET is mechanical grinding followed by melting and reforming.^8^ Such processes lead to a downcycling: each cycle produces a material with fewer mechanical properties of interest, which eventually hampers further recycling.^8^

In recent years, plastic recycling research has gained new momentum with the identification of a PET hydrolase isolated from *Ideonella sakaiensis* capable of degrading PET close to room temperature.^9^ Many other PET hydrolases have been characterized since then, improving catalysis rates, enzyme thermostability, and reaction conditions.^10−15^ Still, most enzymatic protocols struggle with degrading high-crystallinity substrate or performing well under low-pH conditions,^5^ which are the main challenges in reducing the cost of enzymatic PET degradation.^7^

More recently, molecular dynamics (MD) simulations have provided many structural insights into the interactions between PET and many different esterases.^13,16−19^ In addition, other studies have modeled the interaction between carbohydrate-binding modules and PET crystal surfaces to understand the forces driving such interactions, aiming to improve the binding of PET hydrolases onto high-crystallinity regions of PET.^20,21^ At the core of those studies, force field parameters modeling PET nonbonded interactions will determine the accuracy of any prediction. Current force field models available for PET do not capture the complex electronic polarization effects of PET residues, which likely influence the behavior of PET in specific electronic microenvironments, such as enzyme active sites or at PET-solvent interfaces.

Here, we aim to fill this gap by presenting a Drude polarizable model for PET polymer and its derived molecules, along with example cases to probe the performance of our model. Ultimately, we demonstrate how the inclusion of explicit electronic polarization in our model allows for a more complete understanding of the atomistic forces driving solvent interactions and protein binding to amorphous and crystallized PET polymer.

## Methods

### Parametrization of PET-Derived Molecules

The parametrization protocol used to develop our PET model was based on previously published methods used for the development of protein, nucleic acid, lipid, and carbohydrate Drude parameters.^22−30^ Briefly, molecular polarizabilities, dipole moment, water interactions, and dihedral potential scans were used as target data. A detailed description of our protocol is provided as Supporting Information.

**Figure 1:**
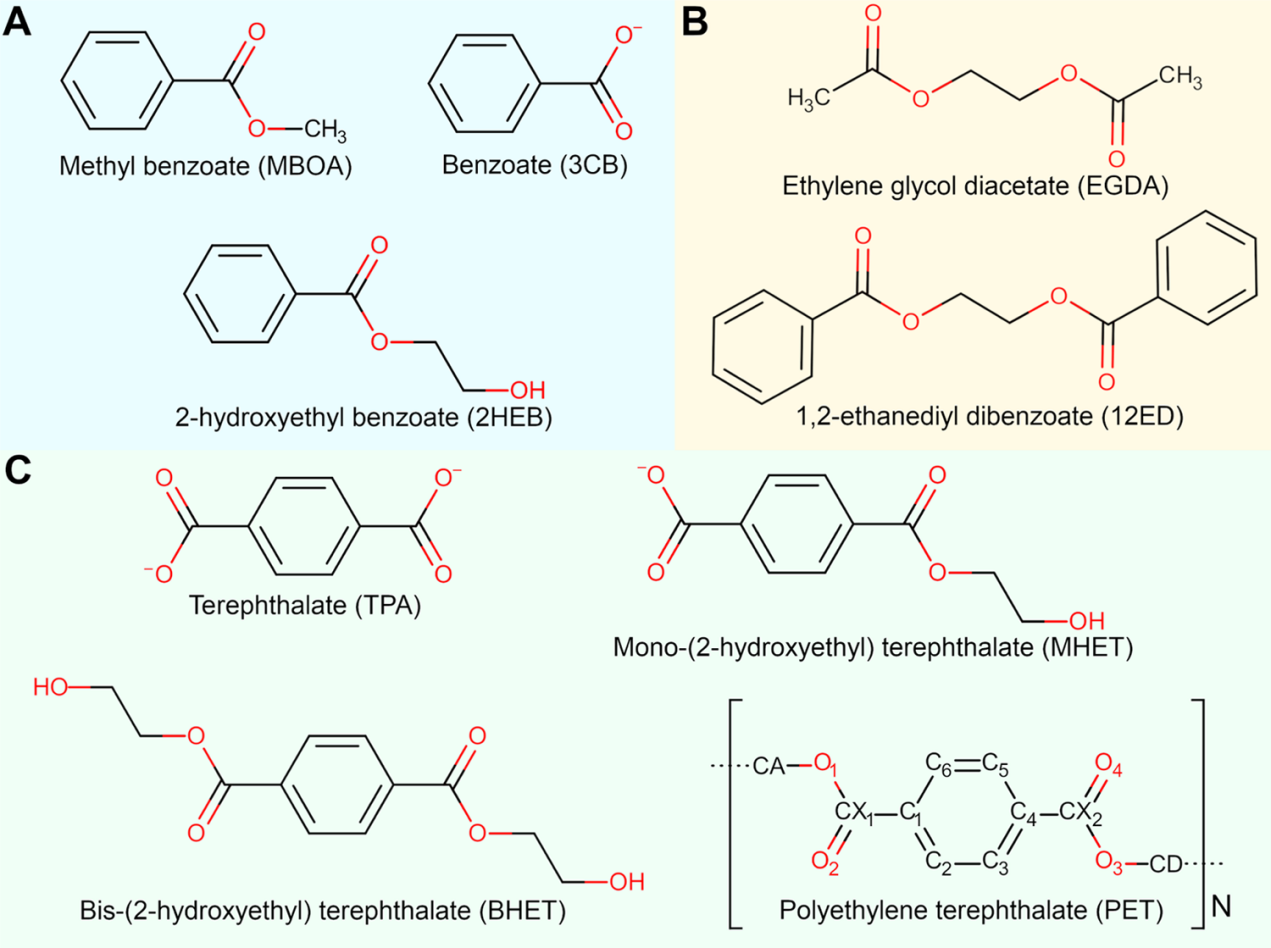
(A) Model compounds used to fit bonded and nonbonded terms for PET-derived molecules; (B) Model compounds used to fit dihedral parameters for the linkage between PET residues; (C) PET-polymer and its derived molecules included in this parameter set.

Here, we used methyl benzoate (MBOA), benzoate (3CB), 2-hydroxyethyl benzoate (2HEB), 1,2-ethanediyl dibenzoate (12ED) and ethylene glycol diacetate (EGDA) as model compounds (Figure 1A,B) to fit new dihedral and nonbonded parameters for the PET polymer and its derived molecules, i.e., terephthalate (TPA), mono-(2-hydroxyethyl) terephthalate (MHET), and bis(2-hydroxyethyl) terephthalate (BHET), as shown in Figure 1C. Atom naming convention for the PET polymer is shown in Figure 1C.

### System Building Protocols

#### Amorphous PET Simulations

To build an amorphous PET material, we used the CHARMM program^31^ to arrange an 18 × 13 array of PET polymer composed by 9-mer chains spaced laterally and vertically to fill a box of 100 Å × 108 Å × 52 Å. CGenFF parameters^32,33^ were used to build the crystal PET chain topology and terminal methylation at both ends of each chain. In total, our simulation box had 234 PET polymer chains. The energy of the system was minimized via 500 steps of steepest descent minimization followed by 500 steps of adopted-basis Newton–Raphson minimization.

To induce glass transition amorphization of our system, we based our approach on the work of Sahihi et al.^34^ and performed a series of 1 ns simulations in OpenMM^35^ to increase the temperature of the system from 300 to 800 K in an NVT ensemble, in increments of 50 K every simulation. To cool down the system, the reverse strategy was applied under an NPT ensemble while applying a pressure of 50 bar via a Monte Carlo Membrane barostat^36,37^ in which *x*- and *y*-axes were scaled anisotropically while the *z*-axis was allowed to vary independently. In this barostat scheme, a Monte Carlo algorithm is used to periodically attempt rescaling of the box vectors. A final NPT simulation was carried out at 300 K and 1 bar for 10 ns to allow for equilibration and relaxation of the amorphous PET material model. A complete description of simulation parameters used for all additive (i.e nonpolarizable) systems is provided below. Finally, amorphous phase was checked by calculating end-to-end vectors of all PET chains by extracting the *x*-, *y*-, and *z*-axis components of the distance between residue 1 and residue 9 termini methyl carbons. Bond vectors were calculated similarly using the carbons of the ethylene glycol (EG) linkage (CD and CA, as shown in Figure S1C) of residue N and N+1.

The equilibrated coordinates were then converted to the Drude model in CHARMM and further equilibrated for 500 ps. Position restraint forces were applied to heavy atoms with a force constant of 1000 kJ mol^–1^ nm^2^. Periodic boundary conditions were applied in all dimensions and pressure was kept at 1 bar via the Monte Carlo Membrane barostat described above. Unbiased production simulations were carried out for both the nonpolarizable and Drude systems for 500 ns under an NPT ensemble for data collection, saving coordinates every 10 ps. A complete description of Drude conversion and simulation parameters used for all polarizable systems is provided below.

Additionally, the final coordinates of our Drude PET model material were used to further study the electronic and structural properties of an amorphous PET slab in vacuum and water. To create our slab model in vacuum, we increased the *z*-axis dimensions of the simulation box by 20 Å on both sides, while keeping the *x*- and *y*-axis box dimensions constant to preserve interactions across the periodic boundaries. To do so, molecules crossing the periodic boundary conditions were reimagined to preserve the PET slab structure. To build the solvated system, we converted the system to its nonpolarizable counterpart by deleting the Drude oscillators and lone pairs and solvated the enlarged box with CHARMM-modified TIP3P water^38,39^ to fill the open volume. The energy of the system was minimized, followed by equilibration for 500 ps using the protocol described above. The solvated, nonpolarizable system was converted to Drude following the protocol described below. Equilibration was carried for both *in vacuo* and solvated Drude systems for 500 ps using the semiisotropic Monte Carlo Membrane barostat mentioned above. For the *in vacuo* system, the Monte Carlo Membrane barostat with a fixed *z*-axis was used to impose a vacuum layer. Production runs for each system were carried for 100 ns and coordinates were saved every 10 ps.

### Crystal PET Unit Simulations

To build a crystal PET unit, we generated the coordinates of a small crystal sample by replicating the crystal packing information obtained by Tse and Mak^40^ using Avogadro.^41^ The system comprised 72 PET polymer chains, arranged into 6 crystal layers such that each layer contained 12 repeats of hexameric chains. In doing so, we sought to model a thin PET crystal unit while preserving the structural and spatial organization between polymer chains. This approach allowed us to simultaneously study the electronic properties of the crystal interfaces and the possible effects caused by the presence of crystal edges created by the chain end groups.

The system was then parsed by CHARMM and both ends of each PET chain were methylated. An initial box of 90 Å × 90 Å × 90 Å was built around the crystal unit to generate the *in vacuo* model. TIP3P water molecules were inserted into the box using CHARMM to generate the solvated system. The energy of both systems was minimized via 500 steps of steepest descent minimization followed by 500 steps of adopted-basis Newton–Raphson minimization. Equilibration was carried in OpenMM for 1 ns while a position restraint was applied on all heavy atoms with a force of 500 kJ mol^–1^ nm^2^.

The equilibrated coordinates were then converted to the Drude model in CHARMM as described below. Finally, Drude systems were equilibrated for 1 ns. An isotropic Monte Carlo barostat was used to maintain the pressure at 1 bar by attempting box scaling every 25 integration steps. Position restraints were applied to heavy atoms during equilibration with a force constant of 800 kJ mol^–1^ nm^2^. For the solvated and *in vacuo* systems of both nonpolarizable and Drude models, unbiased simulations were performed for 100 ns, saving coordinates every 10 ps. While the production simulations of solvated systems were carried out under an NPT ensemble, the *in vacuo* simulations employed an NVT ensemble. In all systems, a weak position restraining force of 50 kJ mol^–1^ nm^2^ was applied to the bottom layer of the crystal unit (Layer −3) to facilitate postprocessing reimaging and analyses.

### PET-Protein Binding Simulations

We tested the performance of our PET parameters when modeling protein binding events by simulating the binding of a hevein, a lectin with chitin-binding specificity from *Hevea brasiliensis*,^42,43^ to the PET crystal unit described above. To do so, we simply placed the hevein such that its center-of-mass was about 14 Å away from the crystal surface, with its chitin-binding domain facing the PET residues. The protein topology was generated using CHARMM36m force field.^44^ The system was subsequently solvated in CHARMM with TIP3P water molecules and neutralized with a total ionic strength of 0.15 M of KCl, including counterions. Equilibration of the nonpolarizable system, conversion to Drude, and production runs were carried out as described below. For the Drude systems, the protein topology was generated using the latest Drude-2019 parameters.^45^

### General Nonpolarizable and Polarizable Simulation Protocols

We used the same nonpolarizable and polarizable simulation parameters for the different systems detailed above. The short-range Lennard-Jones forces were switched smoothly to zero from 10 to 12 Å and electrostatic forces were calculated beyond a real-space cutoff of 12 Å via PME.^46^ An integration time step of 2 fs was used with a Langevin integrator maintaining a temperature of 300 K. Different schemes of the Monte Carlo barostat algorithm were applied as described above for each system. Coordinates were saved every 10 ps.

For our Drude simulations, equilibrated coordinates of each system was converted to the Drude model using the CHARMM program. After, Drude oscillators were relaxed via energy minimization in CHARMM by employing 1000 steps of steepest descent minimization, followed by 500 steps of adopted-basis Newton–Raphson minimization. For solvated systems, TIP3P water models were converted to SWM4-NDP model.^47^ Equilibration of each system was subsequently carried out using OpenMM^48^ using the Langevin integration algorithm^49^ and a 1 fs time step. A “hard wall” constraint^50^ was enforced on Drude oscillators at 0.2 Å to prevent polarization catastrophe. A dual Langevin thermostat was used to maintain the average temperature of real particles at 300 K while Drude oscillators were kept at a relative temperature of 1 K. Different schemes of the Monte Carlo barostat were applied to each system as described above. The Lennard-Jones potential was also switched to zero from 10 to 12 Å while electrostatic forces beyond a real-space cutoff of 12 Å was calculated via PME, as is conventional for the Drude force field.^51^ Coordinates were saved every 10 ps.

### Electrostatic Potential Calculations

For each of the systems, we used the last frame of production simulation and relaxed the Drude oscillators via energy minimization in CHARMM, as described above for Drude conversion. We then generated the Connolly surface of the PET molecules (either amorphous slab or crystal array) using the MSMS software^52^ with a probe radius of 1.5 Å. Finally, we calculated the electrostatic potential (*V*(*r*_p_)) exerted by PET residues at each vertex of the Connolly surface (*r*_p_) defined by MSMS according to eq 1:
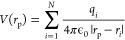
1in which *q*_*i*_ is the partial atomic charge (Drude oscillators and lone pairs included) and *r*_p_ is the position of the particle. The vacuum permittivity (ϵ_0_) was set to 8.8541878128 × 10^–12^ F m^–1^. By using this approach, we were able to account for heterogeneous electronic microenvironments at the PET surfaces, allowing for a better description of the electrostatic potential.

## Results and Discussion

### Development of PET Model Parameters

Accurate modeling of molecular electrostatic properties is one of the most important aspects of the Drude force field. As shown in Table 1, the optimized parameters yielded gas-phase total dipole moments and their components that were in good agreement with their QM target values. An interesting challenge arose when fitting the dipole components of MBOA and 2HEB due to the asymmetric electron density distribution on the ring carbons as a consequence of their proximity to the ester group. Given the rotation about the ester carbon-ring carbon bond, we averaged the ESP charge density between *ortho* and *para* ring carbons, yielding a smaller dipole moment, which was used as target in our fitting protocol.

**Table 1:** QM and Drude Dipole Moments and their Vector Components (in D) for Model Compounds and PET-Derived Molecules

	QM				Drude			
compound	μ_*x*_	μ_*y*_	μ_*z*_	|μ|	μ_*x*_	μ_*y*_	μ_*z*_	|μ|
MBOA	0.43	1.77	0.00	1.82	0.46	–1.44	0.00	1.51
3CB	9.44	0.00	0.00	9.44	9.38	0.00	0.00	9.38
2HEB	0.40	–3.62	–0.72	3.71	–0.09	–3.37	–0.70	3.45
BHET	0.00	0.00	0.00	0.00	0.00	0.00	0.00	0.00
MHET	19.21	0.64	0.18	19.22	19.86	0.83	0.15	19.88
TPA	0.00	0.00	0.00	0.00	0.00	0.00	0.00	0.00
12ED	–0.01	–0.03	0.17	0.17	0.00	–0.02	0.12	0.12
EGDA	0.00	0.00	0.00	0.00	0.00	0.00	0.00	0.00

The symmetrical nature of BHET and TPA yield gas-phase dipole moments of zero. As their parameters were transposed directly from 2HEB and 3CB, we can also assess the quality of these parameters by evaluating MHET, which was also built by transposing parameters from 2HEB and 3CB. As shown in Table 1, the QM *x*-, *y*- and *z*-axis dipole moment components for MHET were 19.21, 0.64, and 0.18 D, respectively, while the Drude force field yielded 19.86, 0.83, and 0.15 D for the same components. As such, the parameters are transferable and yield good agreement with QM target values.

The ability to electronically respond to changes in the surrounding electric field was evaluated by calculating gas-phase molecular polarizabilities. As shown in Table 2, the final Drude parameters yielded polarizability tensors in almost perfect agreement with QM target data for all model compounds and PET-derived molecules. Exceptions were the linkage molecules EGDA and 12ED, for which our parameters yielded errors of 4.51 and 10.69% for α_iso_ for 12ED and EGDA, respectively. This result is most likely due to the absence of these molecules in the data set used to fit electronic parameters. For EGDA, specifically, parameters were transposed from 2HEB, which probably influenced the polarizability due to its bulky aromatic ring close to the ester group. For more details, see the Supporting Methods.

**Table 2:** Gas-Phase Molecular Polarizability Values (in Å^3^) for Model Compounds and PET-Derived Molecules

	QM (scaled)^a^				Drude			
compound	α_*xx*_	α_*yy*_	α_*zz*_	α_*iso*_	α_*xx*_	α_*yy*_	α_*zz*_	α_*iso*_
MBOA	17.17	13.18	7.28	12.55	17.27	13.12	7.17	12.52
3CB	13.18	11.53	5.69	10.13	13.16	11.67	5.77	10.20
2HEB	19.99	14.58	9.57	14.71	19.76	14.37	9.53	14.55
BHET	31.35	20.35	12.71	21.47	31.12	19.89	13.15	21.39
MHET	23.30	16.20	9.11	16.20	23.10	16.45	9.80	16.45
TPA	17.84	14.13	6.99	12.99	17.81	14.23	6.88	12.98
12ED	35.80	26.06	14.16	25.34	38.41	26.80	14.40	26.54
EGDA	11.60	6.87	8.95	9.14	13.47	6.65	10.58	10.23

a QM polarizabilities were scaled down to 85% of the gas-phase values, except in the cases of 3CB, MHET, and TPA, for which values were scaled to 75% following recommendations in Kognole et al.^53^

Water interactions were evaluated for all model compounds and PET-derived molecules using geometries shown in Figure S1. Values of the minimum interaction distance and interaction energy are listed in Tables S1 and S2. For both BHET and MHET, interaction energies and distances for carbonyl oxygen O4 and the hydroxyl oxygen OE were in very good agreement with QM target data. In case of the ester oxygen O3, interactions were slightly off for the interaction bisecting the lone pairs (O3_180) due to a steric clash caused by a 15° rotation of the ester-ring dihedral that positions the water molecule in this interaction close to the aromatic ring. Moreover, we evaluated the carbonyl oxygen and ester oxygen interactions for 12ED to confirm that our parameters were able to accurately model the linkage between PET monomers, allowing us to model PET polymers of any length.

In addition, we evaluated the anisotropic polarizabilities by scanning water interactions along arcs circling the lone pair axis of the carbonyl oxygen and the ester oxygen using MBOA as reference (Figure S2). For the majority of the data points, interaction energies and distances were in good agreement with QM target data, although the ester oxygen showed larger deviations of ∼1 kcal/mol for some data points and could be improved in the future by more exhaustively targeting QM polarization anisotropies.

**Figure 2:**
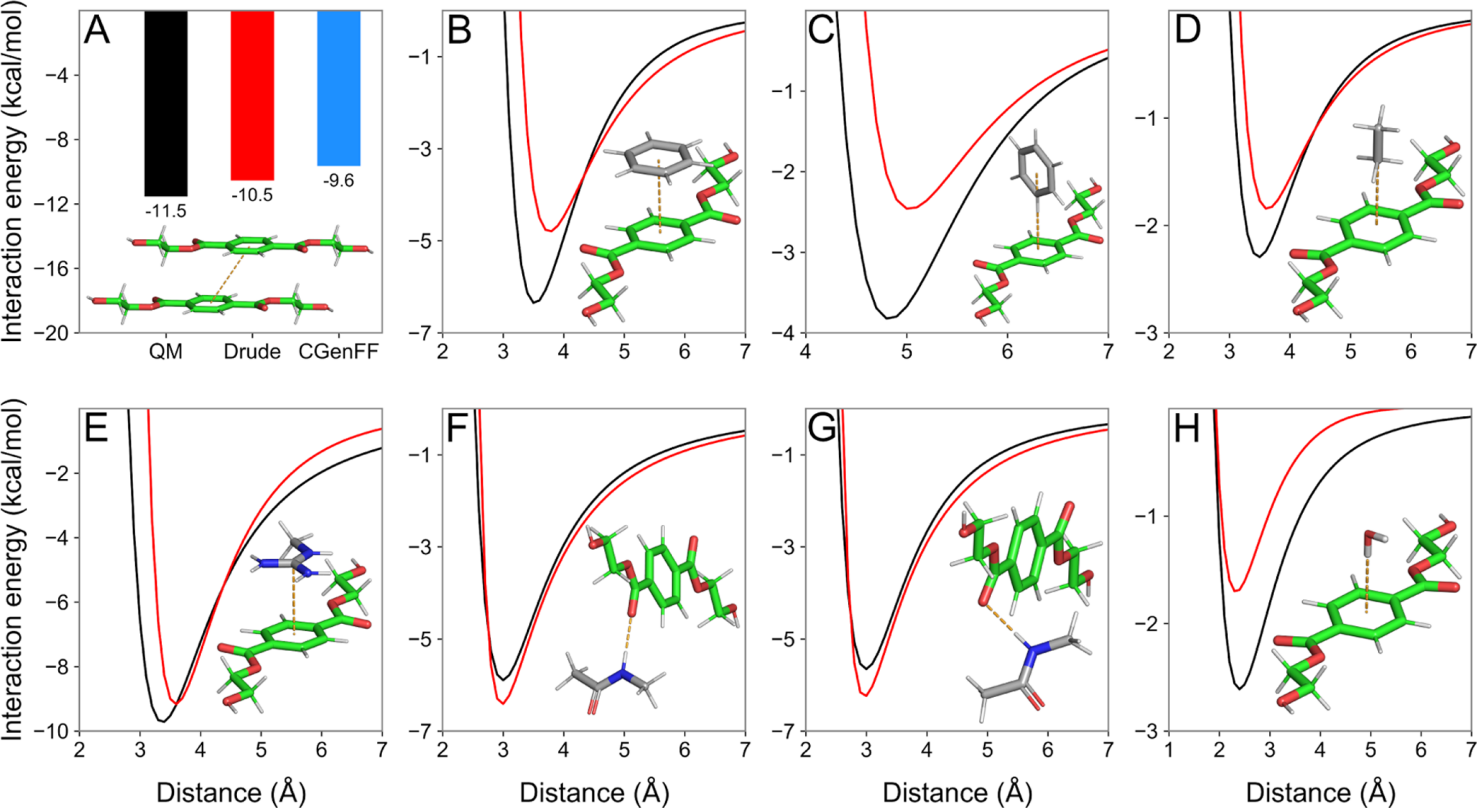
Interaction energy calculations used to probe nonbonded parameters of BHET: (A) Single-point energies for BHET dimer interactions, interaction energy scans for (B) BHET-benzene (sandwich) and (C) BHET-benzene T-shaped π–π interactions, (D) BHET-ethane; (E) BHET-methylguanidinium, (F) BHET-NMA along the carbonyl bond axis, (G) BHET-NMA at the σ-hole angle, and (H) BHET-water. In all panels, black lines represent QM values obtained with RIMP2/aug-cc-pVQZ model chemistry, while red lines represent MM values obtained with the Drude parameters.

As a final assessment of nonbonded interactions of our PET model, we calculated the BHET dimer interaction based on PET crystal packing geometry.^40^ As shown in Figure 2A, our parameters yielded an interaction energy of −10.54 kcal/mol while the QM target value was −11.54 kcal/mol. For comparison, the same interaction using the nonpolarizable CGenFF counterpart of the BHET model yielded an energy of −9.64 kcal/mol, suggesting that electronic polarization might play a role in PET crystal packing energies.

Given the importance of interactions between PET and enzymes,^10,16,18,19,54,55^ we evaluated the interactions between BHET and common protein functional groups to guarantee self-consistency between our parameters and the Drude force field. As shown in Figure 2B,C, benzene (BENZ) stacking interactions with BHET are slightly underestimated, with maximum errors of ∼1.5 and ∼1.4 kcal/mol for sandwich and T-shape stacking interactions, respectively. Ethane (ETHA) interactions (Figure 2D) were also well captured, with a similar interaction energy minimum distance, and well depth difference of less than 0.5 kcal/mol. For methylguanidinium (MGUAN) interactions (Figure 2E), the minimum distance was shifted slightly outward in our Drude model, suggesting a slight repulsion in the MM model relative to the QM calculations for distances shorter than 3.5 Å. Nevertheless, the magnitude of the interaction energy minimum was well captured, suggesting that cation-π interactions with our PET model should be reasonably represented. In addition, we considered *N*-methylacetamide (NMA) interactions with the carbonyl oxygen as a model for the well-known σ-hole interactions observed in PET hydrolases.^19,54^ To do so, we scanned the interaction of NMA along both the carbonyl bond axis and at an angle similar to that observed for σ-hole interactions. In both cases, the energy profiles yielded by Drude were able to reproduce both the QM minimum distance and the well depth accurately (Figure 2F,G). Finally, we also scanned the interaction of a water molecule perpendicular to the BHET ring plane to assess whether solvent interactions would be correctly captured. Once again, our Drude model was able to reproduce the minimum location, although the interaction energy was underestimated by ∼1 kcal/mol, which is within chemical accuracy. Stacking and hydration interactions with the aromatic rings may require additional tuning, for example via off-diagonal Lennard-Jones terms, but this approach would impact other aspects of the force field and is best left to future work.

**Figure 3:**
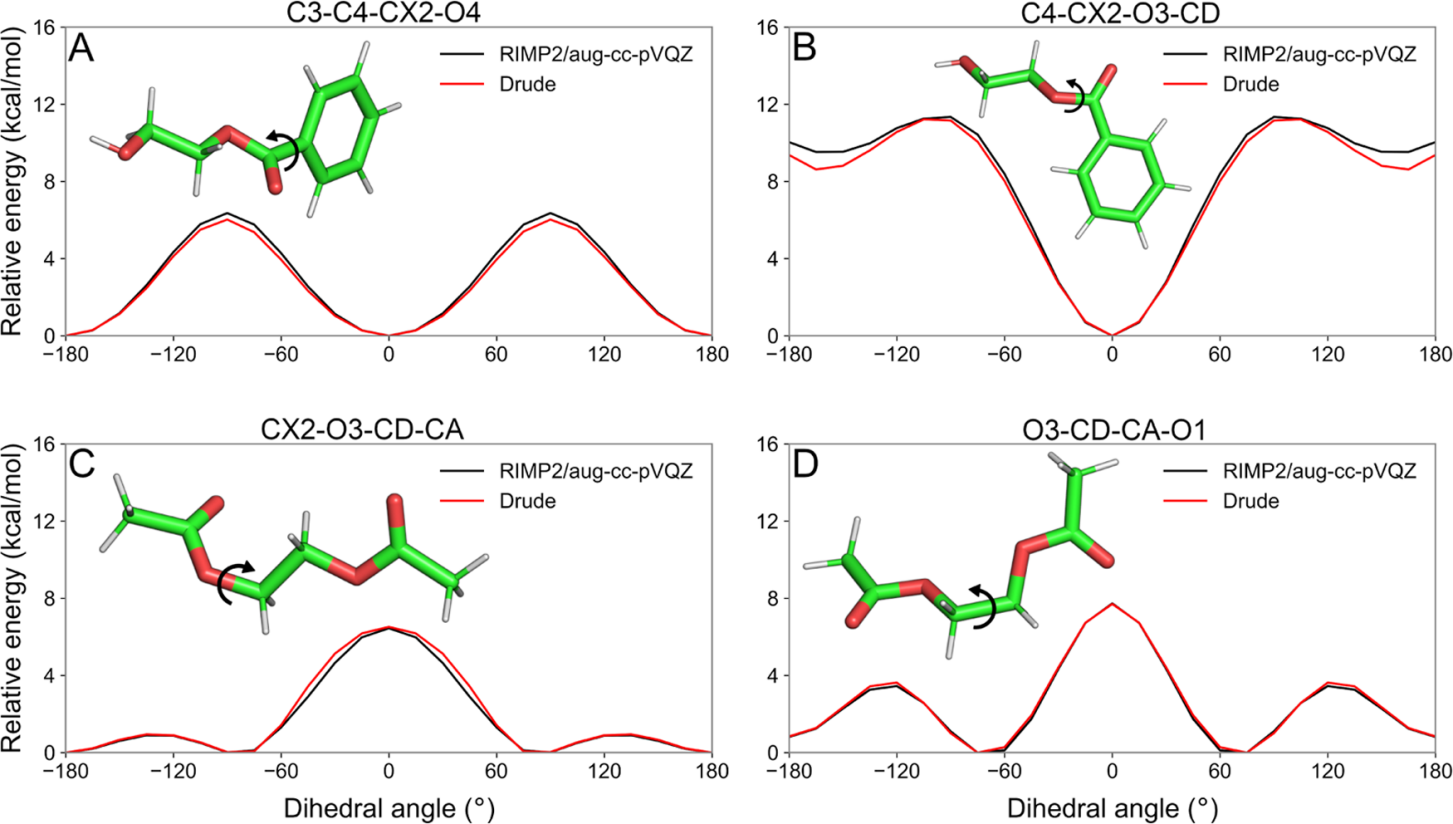
Dihedral scans for torsions of PET polymer that were not previously described by the Drude force field: (A) Ring-ester linkage and (B) rotatable ester bond of of 2HEB, (C, D) dihedrals of EGDA used to fit parameters for the linkage between PET residues.

Finally, we derived new dihedral parameters for torsions that had not previously been optimized for the Drude force field. Careful tuning was required to guarantee that our model would capture the conformational dynamics of PET polymer and its derived molecules. As shown in Figure 3A,B, we used 2HEB to derive parameters for the bond between the ester carbon and the aromatic ring and the ester rotatable bond (defined in Figures S3A,B, respectively). For both torsions, our Drude parameters were able to reproduce the energy minima and barrier heights of ∼6 and ∼11 kcal/mol. For torsions associated with the EG moiety linkage between PET residues, we used EGDA to scan the dihedrals CX2-O3-CD-CA and O3-CD-CA-O1 as defined in Figure S3C,D and shown in Figure S3C,D, respectively. The torsion O3-CD-CA-O1 is commonly defined as Ψ^56,57^ and is directly tied to the *gauche* (Ψ_g_ ∼ 70°) and *trans* (Ψ_t_ ∼ 180°) torsional populations in crystalline or amorphous PET.^56^ In our torsional profiles, two equivalent minima are located at the *gauche* angles with the *trans* configurations ∼0.85 kcal/mol higher, in line with relative energetics proposed by Schmidt-Rohr et al.^56^

The final set of Drude parameters developed in this work for each model compound, PET-derived molecule, the PET monomer itself and polymer termini patches can be obtained at https://github.com/mdpoleto/drude_petpolymer.

### Electronic Properties of Amorphous PET

We tested our parameters by modeling an amorphous PET material to study its structural and electronic properties under our nonpolarizable and polarizable models. At room temperature, amorphous PET has a density of 1.34 g/mL.^58^ In our production runs, the Drude and CGenFF systems produced average densities of 1.218 ± 0.003 and 1.233 ± 0.002 g/mL, respectively (Table 3). A complete density time series are presented in Figure S4. Although we observed a better *in vacuo* BHET dimer interaction energy using our Drude model relative to CGenFF, these results suggest a slight overestimation of Lennard-Jones repulsion for some of the ring atoms, the cumulative effects of which may be leading to an overall increase in intermolecular distances in condensed-phase applications.

**Table 3:** Experimental and Predicted Structural Properties of Amorphous PET Materials

	experiment	Drude	CGenFF
density (g/mL)	1.34	1.218 ± 0.003	1.233 ± 0.002
			
*trans* content	14 ± 5%	17.3 ± 0.4%	14 ± 0.4%
avg |Ψ_t_|	180°	172 ± 5°	172 ± 5°
			
*gauche* content	86 ± 5%	82.7 ± 0.4%	86 ± 0.4%
avg |Ψ_g_|	70°	71 ± 12°	69 ± 12°

Despite this minor deviation in density, our Drude parameters were able to reproduce experimentally observed structural data (Table 3). From our Drude simulations of amorphous PET in a periodic system, we observed an average Ψ_t_ content of 17% corresponding to an average |Ψ_g_| of 71 ± 12°. CGenFF parameters yielded similar Ψ_t_ content (14%) and an average |Ψ_g_| of 69 ± 12°. These results are remarkably close to the experimental average Ψ_g_ content of ∼14 ± 5%, with an average |Ψ_g_| of 70°.^56^ Interestingly, our amorphous PET model demonstrated very little flexibility at 300 K for either model, as observed by the consistency of the *gauche*/*trans* ratio over time (Figure S5). To build upon the well-modeled PET structural properties, future work should test our model in terms of mechanical properties such as elasticity, stress response, and pore formation under the influence of applied electric fields.

To further confirm that our system reached an amorphous phase, we calculated end-to-end vectors of each PET chain in our system (Figure S6). As shown in Figure S6A, the random end-to-end vector orientation in the *xy*-plane suggests a disorganized arrangement of PET chains in our system. In Figure S6B, the distribution of these end-to-end distances shows a peak close to 30 Å, with maximum values reaching 60 Å, which is far from the 100 Å distance that a linear 9-mer chain in crystal phase would adopt, based on crystal data obtained by Tse and Mak.^40^ Similarly, we calculated the bond vector of all PET linkages in our system (Figure S7A,B). The bond vectors projected onto the *xy*- and *yz*-planes also showed random directions, emphasizing the amorphous nature of our system. Taken together, these results and the reproduction of the expected *gauche*/*trans* ratio of an amorphous PET material indicate that we were able to model an amorphous PET sample with the protocol employed here.

**Figure 4:**
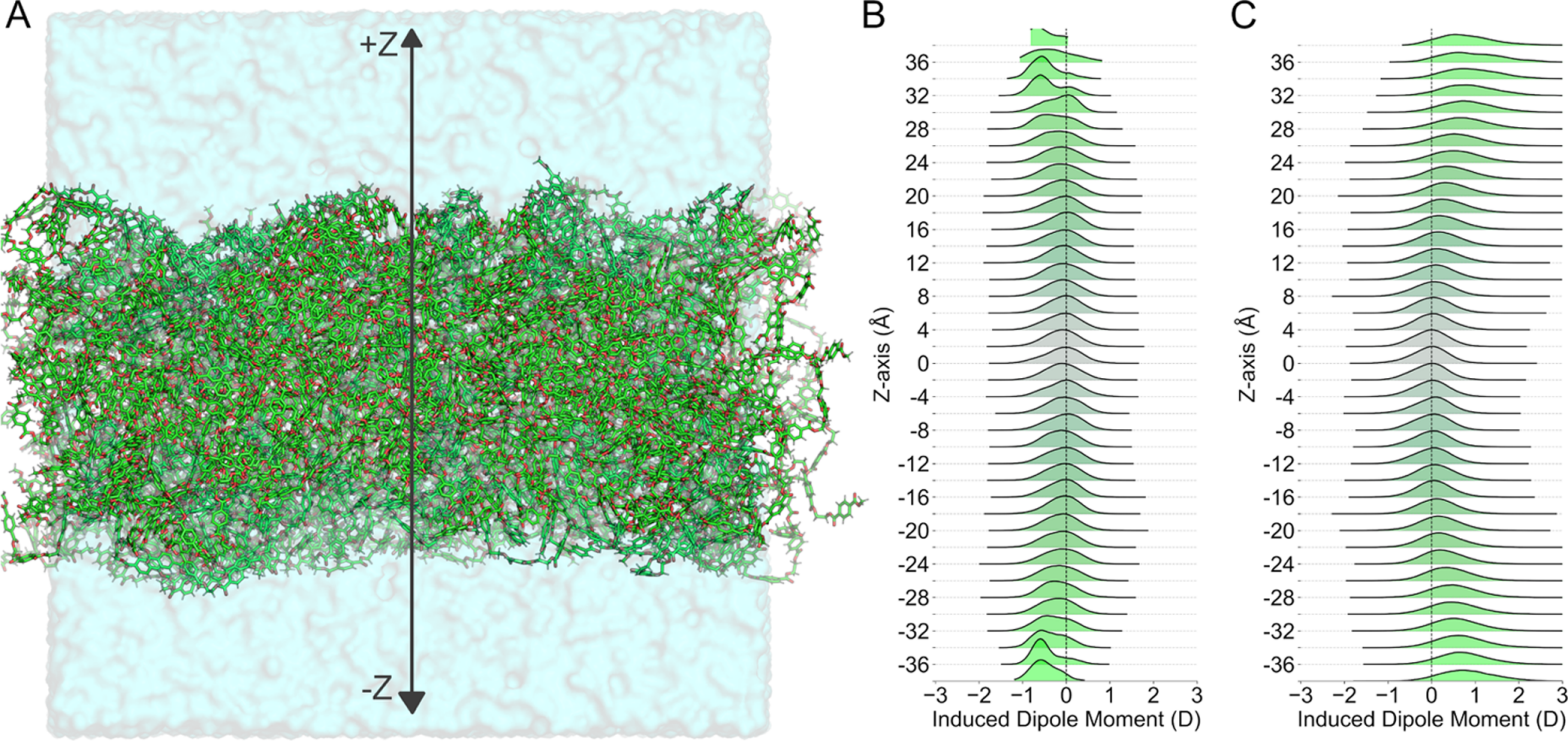
Electronic properties of PET residues in an amorphous slab. (A) Scheme of the orientation of the *z*-axis across the PET slab for the solvated system. Induced dipole moments (in D) were calculated and binned as a function of the PET residue location along the *z*-axis. (B, C) Distribution of induced dipole moments for in vacuum and solvated systems, respectively.

To better understand the dynamics and electronic properties, we performed simulations of our amorphous PET slab model in vacuum and in water (Figure 4A). We calculated the induced dipole moment by subtracting the permanent dipole moment from the total dipole moment of each PET residue in the slab and binned them based on the *z*-axis coordinate of their centers-of-mass. As shown in Figure 4, the induced dipole distribution at the center of the slab is close to zero in both systems, most likely reflecting the cancellation on average of polarization influences due to the unorganized structure of an amorphous phase. Interestingly, the induced dipole moments in vacuum trended toward slightly negative values at slab-vacuum interface (|*Z*| > 25 Å), reaching approximately −1 D in the most exposed monomers (Figure 4B). These results are consistent with the dipole moment decomposition for model compounds shown in Table S3, suggesting that PET residues tend to depolarize when exposed to vacuum conditions.

**Figure 5:**
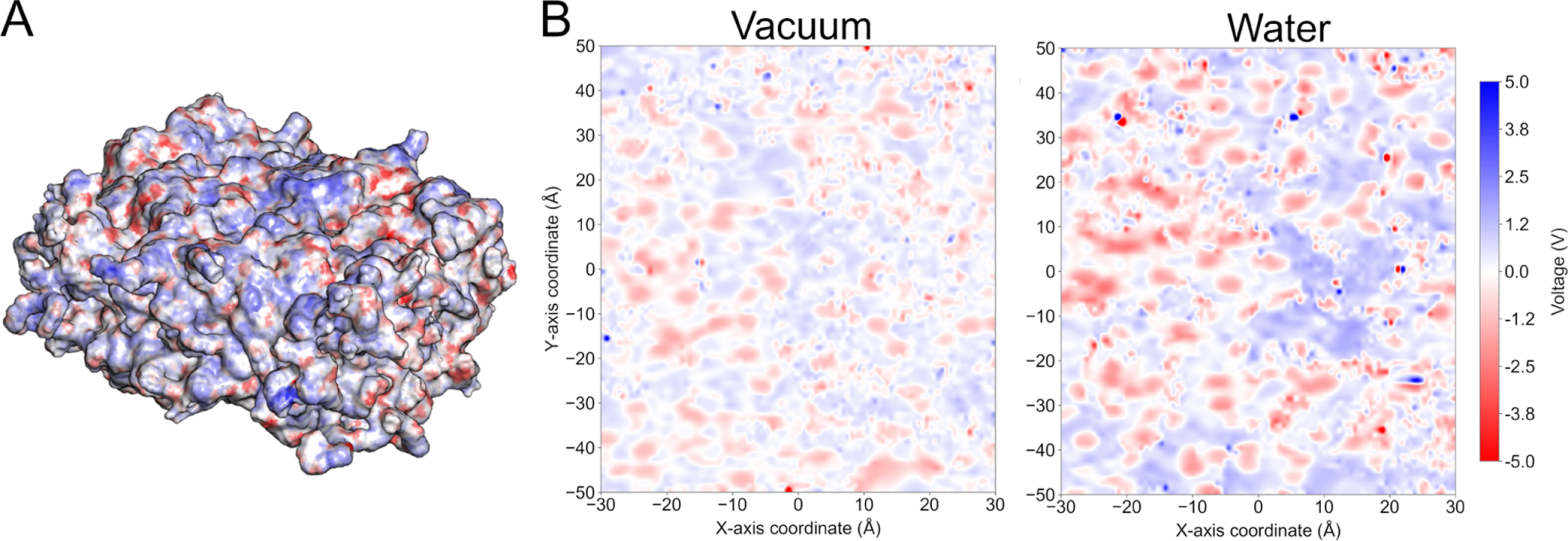
Electrostatic potential (in V) surfaces of amorphous PET slabs. (A) Scheme of a PET slab surface computed by MSMS and colored by electrostatic potential. (B) Potential map for the amorphous PET slab in vacuum and in water.

In contrast, PET residues at the slab interfaces have a positive induced polarization response when exposed to water; that is, their dipole moments increase rather than decrease as in the case of the vacuum systems (Figure 4C). As shown in the broad induced dipole distributions at the interfaces (|*Z*| > 25 Å), PET residues sampled induced dipole values up to 3 D, with an average close to 1 D per residue. The cumulative effect of this polarization response among all residues at the slab-water interface is likely to be relevant when modeling solvent interactions or protein-PET binding interactions. Based on these findings, we set out to further characterize the electrostatic potential surface of the amorphous slab. As shown in Figure 5, the hydration of the slab surface increases the electrostatic potential range sampled in relation to the system in vacuum. These results highlight the electronic plasticity of PET in amorphous phase, which could play an important role when interacting with other solvents, materials and enzymes.

### Electronic Properties of Crystalline PET Arrays

**Figure 6:**
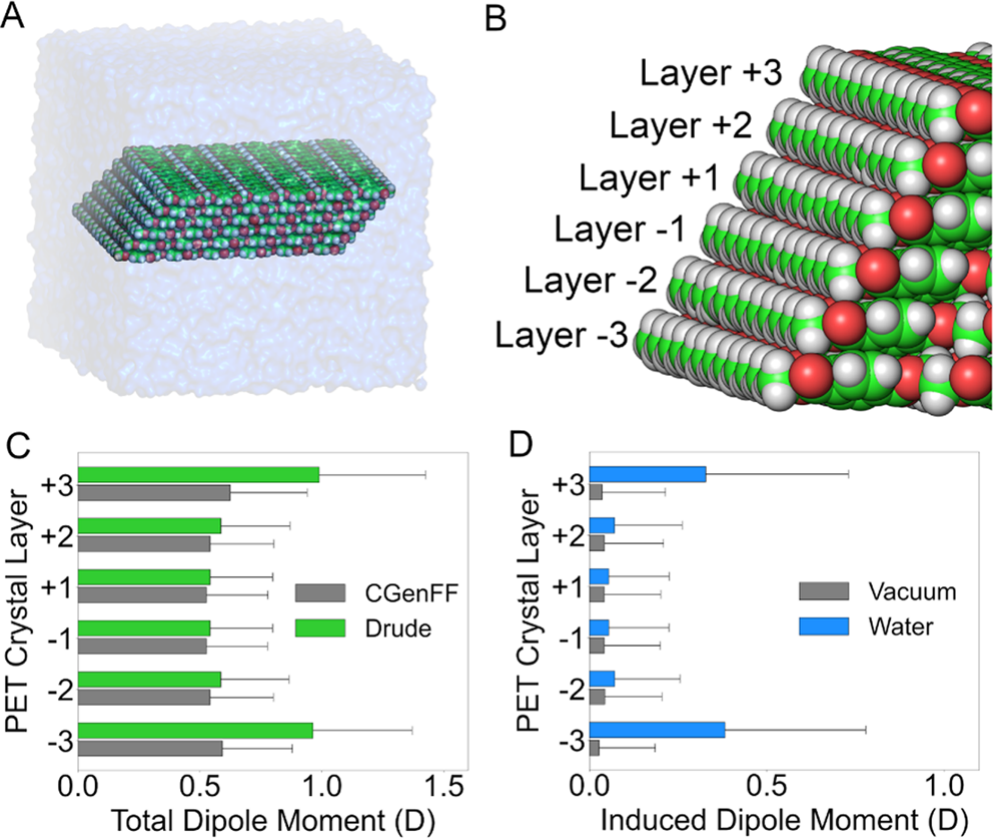
Electronic properties of crystalline PET unit. (A) Rendering of the system containing the crystal PET unit in solution. (B) Scheme depicting the crystal unit layers and associated numbering. (C) Average total dipole moments per layer for nonpolarizable (gray) and Drude systems (green). (D) Average induced dipole moments per layer for the Drude in vacuum (gray) and solvated (blue) systems.

To further test our model, we also built and studied crystal arrays of PET polymer chains in vacuum and in water (Figure 6A). In crystalline PET, the polymer chains align in a repetitive, linear arrangement to form well-ordered layers (Figure 6B), forcing a Ψ_t_ of approximately 180° in all PET linkages and yielding nearly 100% *trans* content.^56^ This same pattern was observed in our simulations in vacuum and in water, with average |Ψ_t_| values of 172 ± 7 and 172 ± 9°, respectively (Figure S8).

To demonstrate the negligible impact of the weak restraint applied to Layer −3 on the PET crystal dynamics, we simulated the Drude system containing the PET crystal unit in solution without the weak restraints. We calculated ring-to-ring distances between the PET chains of each layer (Figure S9A) for the unrestrained and the weakly restrained systems. As shown in Figure S9B, the average ring-to-ring distance for all layers is approximately 6.0 ± 0.2 Å, regardless of whether restraining forces were applied or not. This result suggests that our force field parameters are able to reproduce the PET crystal structure and that our weak restraint potential scheme does not bias the PET crystal dynamics.

As with the amorphous PET slab, we also examined the electronic polarization behavior of PET chains in a crystal unit as a function of their exposure to the solvent. As shown in Figure 6C, the total dipole moments of PET residues in the nonpolarizable system were insensitive to the their position in the crystal layers, producing an average dipole moment of ∼0.5 D in all layers. Meanwhile, in the Drude system, the total dipole moments were increased among the residues at the crystal surfaces, with layers ±3 manifesting a total dipole moment of ∼1 D. As shown in Figure 6D, the increased induced dipole moment of the PET residues at the crystal-water interface (i.e., layers ±3) arises from the water exposure and this effect is confined to the layer in direct contact with the solvent. Moreover, the small induced dipoles observed for the PET residues in the crystal unit in vacuum suggest the inner PET layers produce a very small, localized electronic polarization response on the interfacial layers.

**Figure 7:**
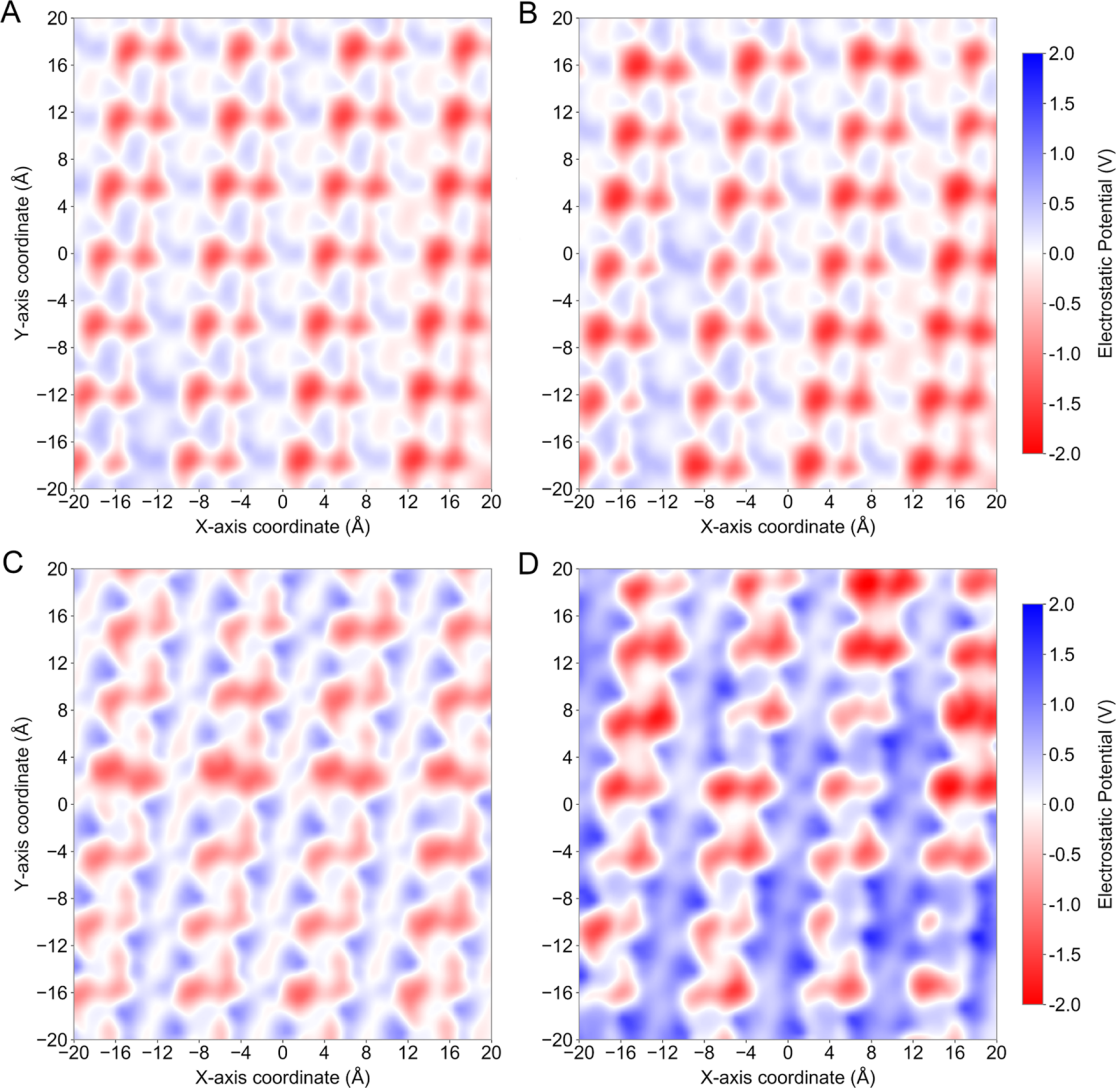
Electrostatic potential (in V) maps at the PET crystal unit surface. (A, B) Potential maps for nonpolarizable systems in vacuum and in water, respectively. (C, D) Potential maps for Drude systems in vacuum and in water, respectively.

We also calculated the electrostatic potential at the unit surfaces for both nonpolarizable and Drude systems (Figure 7). For all systems, regions of negative potential were mostly coincident with oxygen atoms, whereas regions of positive potentials were closer to the aromatic ring atoms. A comparison between the distribution of electrostatic potential values of all systems can be found in Figure S10. For the nonpolarizable systems, the electrostatic potential maps were similar for the systems in vacuum and in water (Figure 7A,B) which is expected due to the lack of explicit electronic polarizability. That is, the partial charges assigned to each atom are invariant and therefore are not sensitive to environmental changes.

Interestingly, the Drude system in vacuum produced a surface potential very similar to the nonpolarizable systems (Figure 7C). An additional shoulder peak at ∼1 V was observed, mostly arising from the aromatic ring atoms. Visually, however, the electrostatic potential maps show small differences, mostly associated with locations of positive potential. In contrast, the Drude system in water produced an electrostatic potential clearly shifted toward positive values, leading to a larger distribution of positive potentials across the crystal surface (Figure 7D). At the same time, the potential was more negative in constricted regions closer to oxygen atoms, reaching values of −2 V, while the strongest potential produced by the Drude system in vacuum was −1.3 V. For comparison, the strongest negative potential produced by the *in vacuo* and solvated nonpolarizable systems were −1.4 and −1.5 V, respectively. Overall, such electrostatic effects are likely to impact the water dynamics at the crystal surface and the desolvation penalty associated with protein binding events to PET crystal regions.

### Electronic Properties of Protein-PET Interfaces

Degrading high-crystallinity PET is still a challenge for current PET hydrolases due to the enzyme binding site structure and poor enzyme adsortion to the PET surface.^5,10,12^ Recently, carbohydrate-binding modules (CBMs) were evaluated as possible anchoring proteins that could enhance the concentration of PET hydrolases at the crystal surfaces and, consequently, facilitate catalysis.^12,14,20,21,59,60^ As such, accurate modeling of protein-PET interactions in multiple media is essential for designing better PET hydrolases or new technologies that could enhance PET degradation.

**Figure 8:**
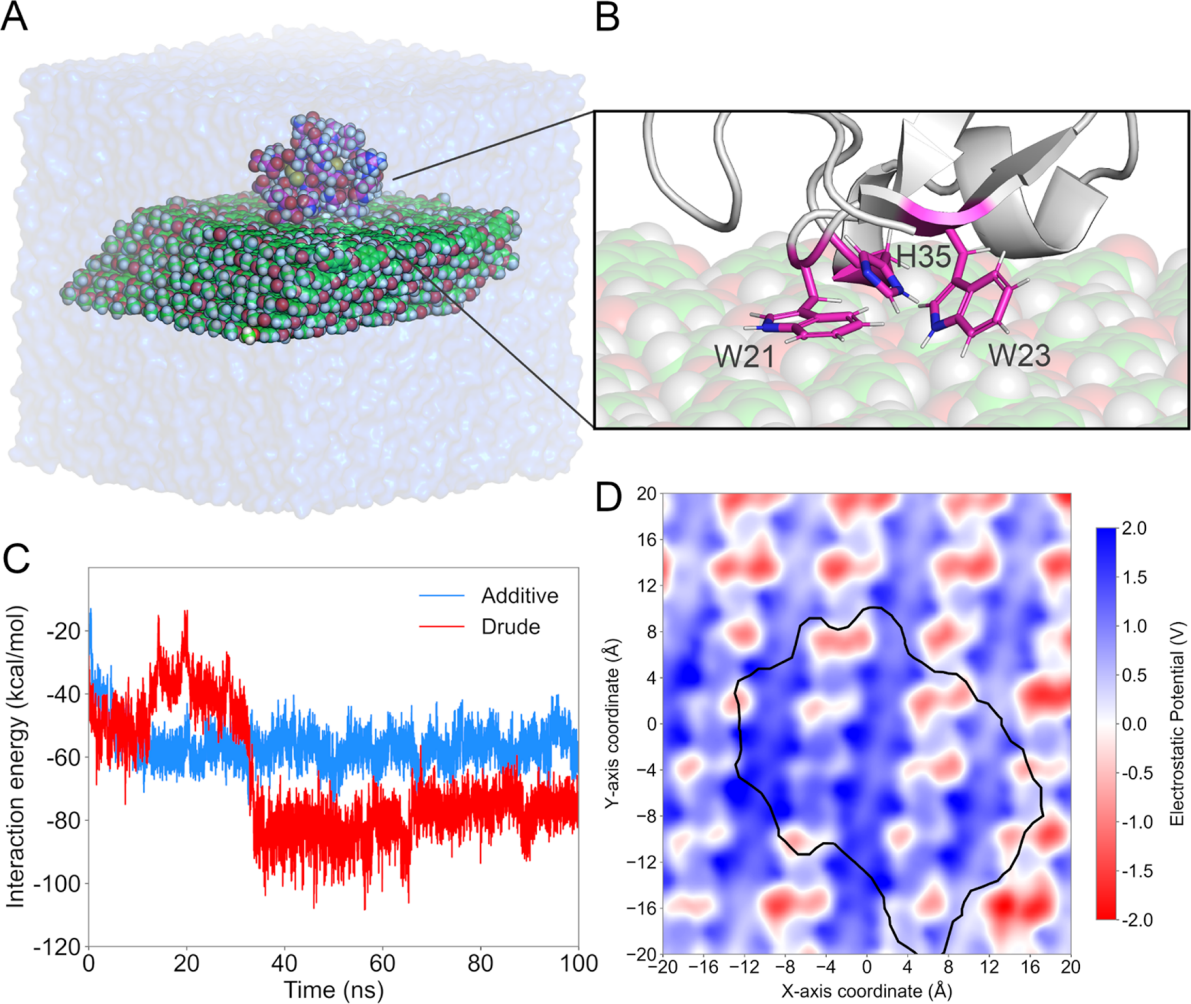
(A, B) Scheme of hevein binding to PET crystal unit, highlighting hevein residues involved in commonly observed interactions. (C) Time series of interaction energies between the PET crystal unit and the hevein protein for nonpolarizable (blue) and Drude (red) systems. (D) Electrostatic potential map (in V) at the PET surface. The black contour indicates the location of the hevein protein bound to the PET surface.

We evaluated the performance of our parameters by simulating the binding of hevein, a chitin-binding protein, to our PET crystal unit (Figure 8A). Throughout both nonpolarizable and Drude trajectories, the residues comprising the chitin-binding domain interacted closely with the PET surface. Residues W21, W23, and H35 engaged in sandwich and T-shaped π–π stacking interactions with PET residues (Figure 8B), while other polar and charged residues transiently interacted with PET residues through water-bridged interactions. Over the course of our nonpolarizable simulation, the hevein rapidly bound to the PET surface, although the same hydrophobic interactions were more intermittent. In our Drude simulation, however, the chitin-binding domain rearranged itself in the first 30 ns of the simulation, better positioning W21 and W23 to promote interactions with the PET crystal surface. Such dynamic events can be drawn from the time series of interaction energies between the hevein and the PET surface (Figure 8C). By the end of the simulations, the Drude systems yielded an interaction energy more than 20 kcal/mol stronger than the nonpolarizable system, reflecting the role of electronic polarization in dictating such protein-PET binding events.

Finally, driven by the difference in interaction energies, we calculated the electrostatic potential at the PET crystal surface when bound to the hevein protein. As shown in Figure 8D, the PET residues bound to the hevein were polarized, increasing the electrostatic potential at the PET surface directly in contact with the protein. This increase is clearly demonstrated in Figure S11 by the positive shift of the electrostatic potential distribution in the system bound to the hevein protein relative to the PET crystal unit in water (Figure 7D). These results suggest that electrostatic interactions between PET crystal surfaces and CBMs could be further explored to design better PET-binding modules.

## Conclusions

Here, we have described the development of a Drude polarizable force field for PET polymers and its derived molecules. The parameter fitting protocol employed here led to accurate modeling of molecular polarizabilities, dipole moments, and water interactions by targeting QM data. Interactions between BHET and protein functional groups were evaluated, showing good agreement between gas-phase QM interaction energies and our Drude model. Lastly, new dihedral parameters were fit to QM configurational energy profiles, allowing an accurate reproduction of energy minima and barriers separating important configurations.

Our parameter set was further tested by modeling amorphous PET. Structural properties of our amorphous model revealed a slight underestimation of PET density, but a very good agreement of *gauche* and *trans* populations in relation to experimental data. By further studying the electronic properties of a PET amorphous slab, we demonstrated the electronic polarization response of PET residues as a function of their exposure to aqueous solvent. We observed an average induced dipole moment of ∼1 D per residue for the areas most exposed to the solvent, with a maximum value of ∼3 D being sampled.

We then tested our parameter set by modeling PET crystal units in different environments. We demonstrated how the most exposed layers of a PET crystal electronically respond to water, yielding an average total dipole moment of ∼1 D per residue in the interfacial layers. In contrast, the inner layers contribute very little to any electronic modulation of the most exposed layers. Moreover, we calculated the electrostatic potential surface of the crystal PET unit, allowing us to map and quantify how solvent molecules modulate the elecrostatic properties of the PET crystal surface.

Finally, we showcase the binding of a hevein to a crystal PET unit, allowing us to identify interacting protein residues and demonstrate the role of electronic polarization in driving such interactions, which led to an average interaction energy between hevein and PET surface almost 20 kcal/mol stronger than in the nonpolarizable model. Lastly, we mapped and quantified how hevein binding to the PET crystal unit modulates the electrostatic potential surface, which could be a strategy used to design better PET binders. Together, these results represent the first extension of the Drude polarizable force field to synthetic polymers. We demonstrated how important the inclusion of explicit electronic polarization of materials is in describing PET material properties and how these outcomes can help designing more efficient (bio)technologies to tackle the plastic pollution crisis.

## Data Availability

To reproduce the work presented here, the Drude polarizable topologies and parameters for the PET polymer and model compounds can be obtained in CHARMM format at https://github.com/mdpoleto/drude_petpolymer.

## Supporting Information Available

Additional text, three tables, and eleven figures are provided that detail force field parametrization protocol, water interaction scheme and values, electronic polarization anisotropy analysis, dihedral definitions, density time series of amorphous PET material, time series of *gauche* and *trans* content of amorphous PET material, electrostatic potential surface maps of amorphous PET, dihedral distribution for crystal PET unit, distribution of electrostatic potentials of crystal PET surfaces in different conditions, end-to-end distance vectors and bond vectors of the amorphous system, and average ring spacing in PET crystal unit in solution (PDF)
