# Supplementary material for: Structural and Electronic Properties of Poly(ethylene terephthalate) (PET) from Polarizable Molecular Dynamics Simulations
Source: Macromolecules. 2024 Nov 8;58(1):403–14. doi: 10.1021/acs.macromol.4c02109 (PMC11741139; doi:10.1021/acs.macromol.4c02109)
Supplement: Supplementary file 1 — ma4c02109_si_001.pdf [file ma4c02109_si_001.pdf]

SUPPORTING INFORMATION

Structural and electronic properties of polyethylene terephthalate (PET) from polarizable molecular dynamics simulations

Marcelo D. Polêto*,† and Justin A. Lemkul*,†,‡

†*Department of Biochemistry, Virginia Tech, Blacksburg, VA 24061, United States*

‡*Center for Drug Discovery, Virginia Tech, Blacksburg, VA 24061, United States*

E-mail: mdpoleto@vt.edu; jalemkul@vt.edu

Supporting Methods

Parametrization protocol

Parametrization of model compounds

First, optimized QM geometries for each model compound were obtained at the MP2/6–31+G* level of theory using Gaussian09.[1] Parameters of model compounds already incorporated in the Drude-2019 parameter set were used initial guesses, as needed. Positioning of lone pairs was based on the closest chemical analog for carboxylic acid and ester compounds available in the parameter set. All molecular mechanics calculations were carried out using the CHARMM program.[2]

Atomic partial charges, atomic polarizabilities, and Thole scaling factors were fit to reproduce gas-phase dipole moments obtained at the MP2/6-31+G* theory level using Gaussian09 and molecular polarizabilities obtained at the RIMP2/aug-cc-pVQZ theory level using Psi4.[3] The fitting procedure was based on a Monte Carlo/simulated annealing (MC/SA) protocol.[4] The error function was defined as the root-mean-square difference between QM and Drude target properties, including the total dipole moment and individual x-, y-, and z-dipole moment components, as well as the isotropic molecular polarizability and the diagonal components of the molecular polarizability tensor. Following the standard Drude parametrization protocol,[5,6] molecular polarizabilities of neutral and negatively charged compounds were scaled down to 85% and 75% of their QM values, respectively, to account for the distortion of the electron clouds in solution.[7]

Solute-water interaction energies from QM were also targeted during parameter fitting following the standard Drude parametrization protocol. Geometries of model compounds were kept fixed in their QM-optimized geometry while water molecules were positioned around the hydrogen-bond donors and acceptors. Optimum interaction distances of water molecules were calculated at the MP2/6–31+G* level of theory while constraining the water geometry to the SWM4-NDP model[8] to allow direct comparisons. Interaction energies were

obtained via single-point RIMP2/aug-cc-pVQZ calculations using counterpoise correction to account for basis set superposition error (BSSE).[9,10] Corresponding molecular mechanics calculations were carried out by using the QM-optimized solute-water dimer configuration.

Lone pair positioning and anisotropic atomic polarizabilities were evaluated by scanning water interactions along the lone pair axis using the same protocol described above, in which the QM optimal binding distances and interaction energies were used as reference.[11] An extra interaction scan was done along the axis orthogonal to the lone pair axis.

Several iterations of the MC/SA fitting protocol followed by testing the electrostatic parameters, and the final electrostatic parameters were accepted once the error between QM target properties were below 5% for dipole moments and molecular polarizabilities and overall below 0.5 kcal mol^{-1} for water interaction energies.

New torsional parameters were obtained for the benzene-ester dihedral present in MBOA and 2HEB and the benzene-carboxylate present in 3CB. Torsions were scanned from -180° to 180° in intervals of 15° with the initial geometry built by CHARMM. Geometry optimization of each configuration was carried out using the MP2/6-31+G* level of theory while allowing other degrees of freedom to relax, followed by single-point energies obtained using RIMP2/aug-cc-pVQZ model chemistry. Molecular mechanical calculations were carried out in CHARMM while parameter fitting was carried out using the MC/SA protocol and the error function was defined as the root-mean-square difference between QM and Drude potential energy profiles. MC moves were performed using the dihedral force constants as parameter space.

Assembling of PET-derived molecules

Once the parameters for MBOA, 2HEB, and 3CB were successfully accepted, we used their chemical groups to assemble TPA, MHET, and BHET. All bonded and nonbonded parameters were recalculated and checked for these 3 molecules as described above to guarantee correct parameter transfer. In addition, BHET dimer interactions were also calcu-

lated to check the self-consistency of our new parameters. PET crystal packing coordinates were obtained from the Cambridge Structural Database (entry 1294009)[12] and a BHET dimer was extracted from it. A single-point energy calculation was carried out in Psi4 at the RIMP2/aug-cc-pVQZ theory level using counterpoise correction to account for BSSE. Single-point energy calculations for Drude and CHARMM36 models were carried out in CHARMM.

Interaction scans between BHET and common protein functional groups were also evaluated to assess self-consistency between the new PET parameters and the Drude-2019 parameter set. We evaluated interactions between the carbonyl oxygen and N-methylacetamide (NMA), hydrophobic interactions between BHET and benzene (BENZ) for both $\pi - \pi$ sandwich and T-shape stacking interactions, cation-π interactions between BHET and methylguanidinium (MGUA). To do so, we used the BHET QM-optimized geometry and the CHARMM internal coordinate builder to properly build the protein functional groups for scanning. Interaction energies were calculated as described above.

Once the parameters for PET-derived molecules were accepted, our efforts focused on parametrizing the linkage between PET monomers to build polymers. To do so, we used 12ED and EGDA as model compounds for the PET monomer linkages. Nonbonded parameters from 2HEB were transposed to 12ED and EGDA (and adjusted as needed) to guarantee transferability of the new torsional parameters. Afterwards, we scanned the torsions around the CH_2-OCH_2CH_2 and OCH_2-CH_2O bonds in EGDA and fit new torsional parameters as described above.

The final Drude parameters for each model compound, PET-derived molecule, the PET monomer itself and polymer termini patches were compiled and used throughout this work. An additive counterpart model was also compiled using the parameters obtained from CGenFF to facilitate system setup.

Supporting Tables

Table S1: QM and Drude water minimum interaction energies (in kcal/mol) and distances (in Å) for model compounds.

	Interaction	E_{QM}	E_{Drude}	$E_{Drude} - E_{QM}$	R_{QM}	R_{Drude}	$R_{Drude} - R_{QM}$
MBOA	O7_180	-4.57	-4.76	-0.19	2.02	1.97	-0.05
	O7_OOP	-3.38	-3.24	0.14	2.13	2.04	-0.09
	O7_LP1	-5.06	-5.27	-0.21	2.00	1.94	-0.06
	O7_LP1	-5.24	-5.44	-0.20	1.98	1.91	-0.07
	O8_180	-2.69	-2.78	-0.09	2.08	2.01	-0.07
	O8_LP1	-3.00	-2.55	0.45	2.08	1.93	-0.15
	O8_LP2	-2.99	-2.73	0.26	2.08	1.94	-0.14
3CB	O1_180	-12.78	-13.18	-0.40	1.80	1.83	0.03
	O1_OOP	-9.68	-9.59	0.09	1.94	1.94	0.00
	O1_LP1	-13.32	-13.44	-0.12	1.78	1.81	0.03
	O1_BID	-8.47	-8.71	-0.24	3.00	3.00	0.00
2HEB	O4_180	-4.36	-4.46	-0.10	2.04	1.99	-0.05
	O4_OOP	-3.45	-3.14	0.31	2.13	2.05	-0.08
	O4_LP2	-4.50	-4.36	0.14	2.01	1.97	-0.04
	O4_LP1	-4.97	-5.07	-0.10	1.99	1.93	-0.06
	O3_180	-2.00	-2.57	-0.57	2.21	2.22	0.01
	O3_LP1	-3.18	-3.01	0.17	2.06	1.91	-0.15
	O3_LP2	-3.05	-3.10	-0.05	2.06	1.95	-0.11
	OE_180	-3.98	-4.49	-0.51	1.98	1.83	-0.15
	OE_LP1	-4.11	-4.12	-0.01	2.00	1.82	-0.18
	OE_LP2	-3.97	-4.12	-0.15	2.00	1.82	-0.18
	HE_180	-5.32	-5.12	0.20	1.90	1.89	-0.01

Table S2: QM and Drude water minimum interaction energies (in kcal/mol) and distances (in Å) for PET-derived molecules and linkage model compounds.

	Interaction	E_{QM}	E_{Drude}	$E_{Drude} - E_{QM}$	R_{QM}	R_{Drude}	$R_{Drude} - R_{QM}$
BHET	O4_180	-4.21	-4.45	-0.24	1.96	1.99	0.03
	O4_OOP	-3.82	-3.80	0.02	2.07	2.00	-0.07
	O4_LP1	-4.88	-5.07	-0.19	1.99	1.93	-0.06
	O4_LP2	-4.40	-4.40	0.00	2.00	1.97	-0.03
	O3_180	-1.70	-2.47	-0.77	2.22	2.23	0.01
	O3_LP	-3.01	-3.36	-0.35	2.08	1.99	-0.09
	OE_180	-3.98	-4.52	-0.54	1.98	1.83	-0.15
	OE_LP1	-3.94	-4.11	-0.17	2.00	1.83	-0.17
	OE_LP2	-3.99	-4.11	-0.12	2.00	1.83	-0.17
	HE_180	-5.46	-5.15	0.31	1.90	1.89	-0.01
MHET	O4_180	-6.39	-6.64	-0.25	1.96	1.93	-0.03
	O4_OOP	-3.97	-3.72	0.25	2.13	2.03	-0.10
	O4_LP1	-6.50	-6.69	-0.19	1.95	1.91	-0.04
	O3_180	-2.73	-3.44	-0.71	2.37	2.24	-0.13
	O3_LP1	-4.93	-4.67	0.26	2.14	1.97	-0.17
	O3_LP2	-5.07	-4.67	0.40	2.15	1.97	-0.18
	OE_180	-4.45	-4.80	-0.35	1.96	1.82	-0.14
	OE_LP1	-4.65	-4.54	0.11	1.98	1.81	-0.17
	OE_LP2	-4.58	-4.54	0.04	1.99	1.81	-0.18
	HE_180	-3.81	-3.94	-0.13	1.94	1.91	-0.03
12ED	O4_180	-4.36	-4.65	-0.29	2.04	1.97	-0.07
	O4_OOP	-3.45	-3.14	0.31	2.13	2.04	-0.09
	O4_LP2	-4.97	-5.26	-0.29	2.01	1.94	-0.07
	O4_LP1	-4.50	-5.12	-0.62	1.99	1.92	-0.07
	O3_180	-2.00	-2.44	-0.44	2.21	2.08	-0.13
	O3_LP1	-3.18	-2.96	0.22	2.06	1.88	-0.18
	O3_LP2	-3.05	-3.10	-0.05	2.06	1.90	-0.16

Table S3: Total, permanent and induced dipole moments (in debye) yielded by our Drude parameters for each model compound.

	Total	Permanent	Induced
MBOA	1.52	2.43	-0.91
3CB	9.38	12.65	-3.27
2HEB	3.45	4.64	-1.20

Supporting Figures

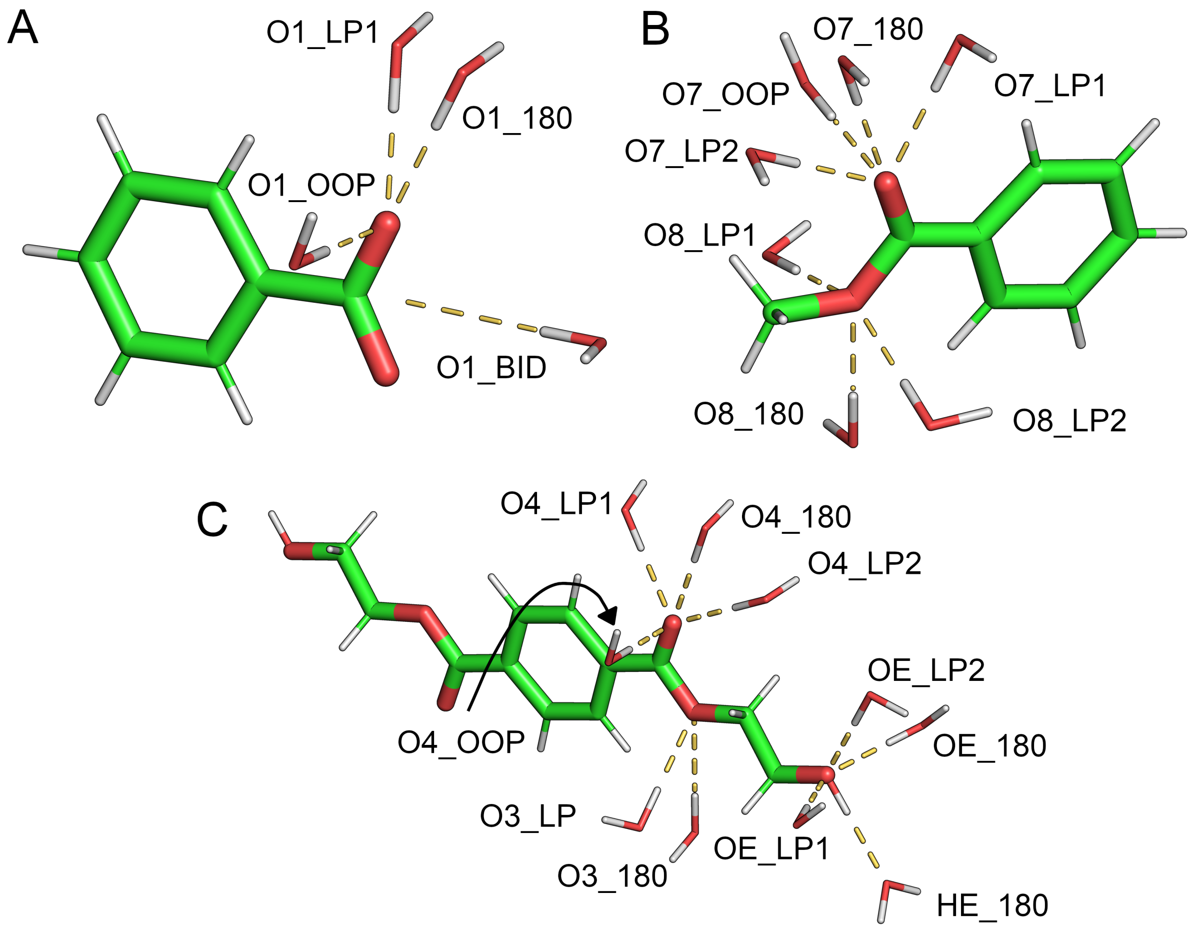

Figure S1: Scheme depicting water interactions used to fit electrostatic parameters in our Drude PET model. A) Orientations used for 3CB. B) Orientations used for MBOA. C) Orientations used for 2HEB, MHET and BHET. Similar orientations following the same patterns were used for EGDA and 12ED.

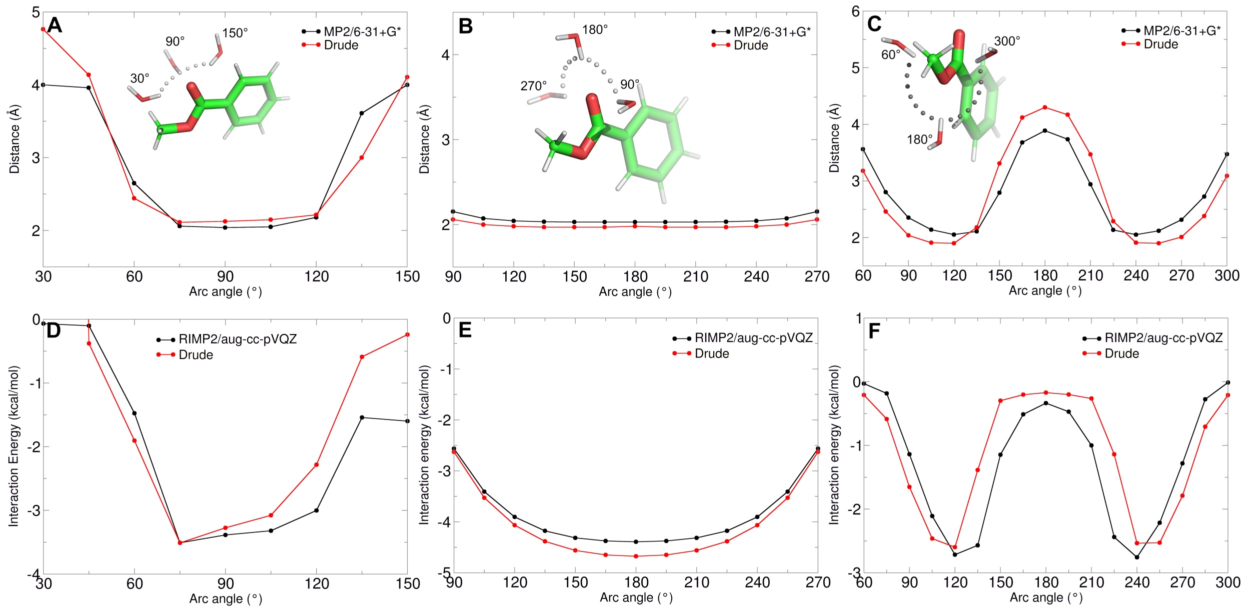

Figure S2: Water interaction scans used to probe polarization anisotropy of our PET model. A, B and C) Optimum binding distances for each scan. QM values (black) were obtained with MP2/6-31+G* model chemistry. D, E and F) Interaction energies for scans shown in A, B and C, respectively. QM values (black) were obtained with RIMP2/aug-cc-pVQZ model chemistry. Drude values are shown in red.

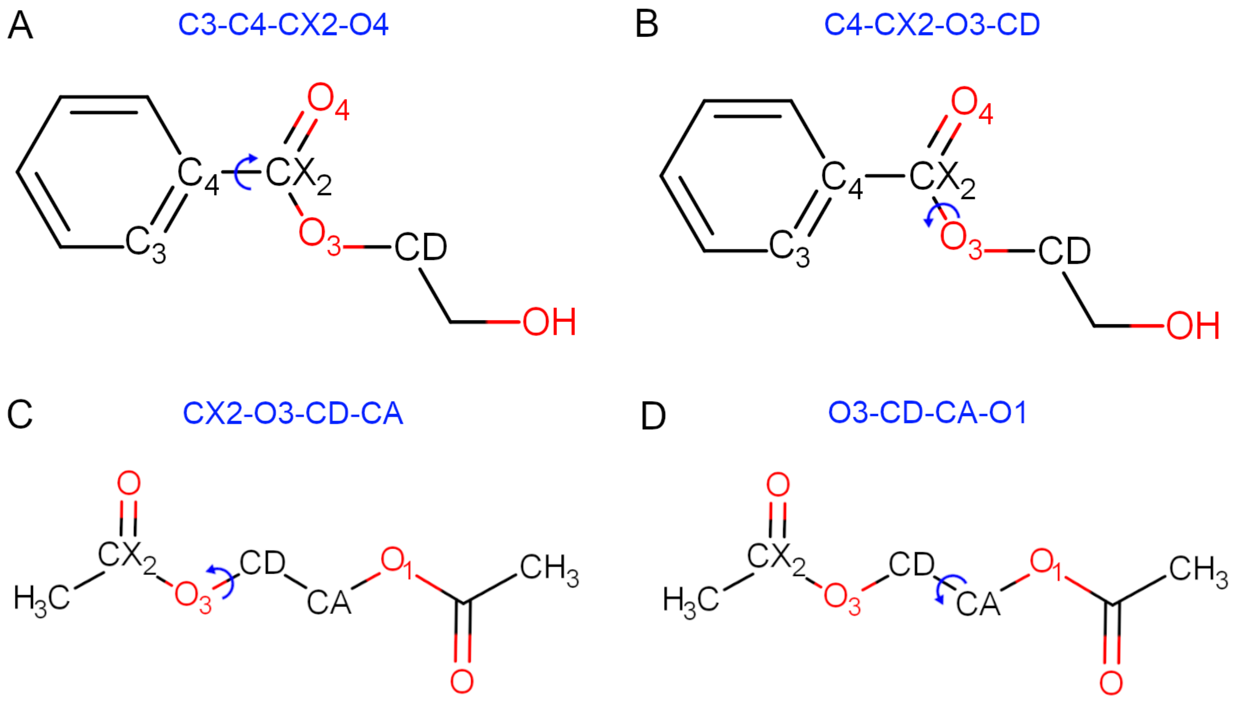

Figure S3: Dihedral definitions used to in parameter fitting. A) Dihedral term of the ester-ring torsion of 2HEB. B) Dihedral term of the ester bond in 2HEB. C and D) Dihedral terms associated to ethylene glycol linkage.

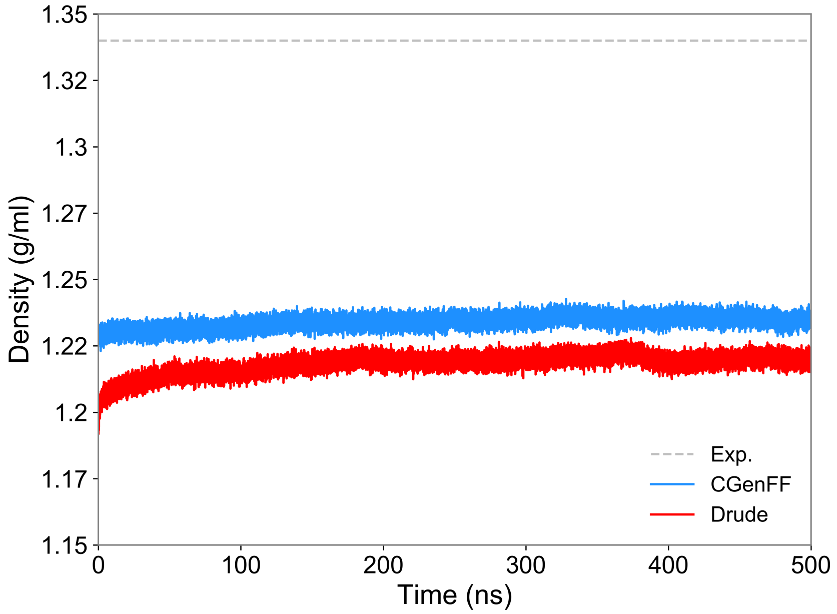

Figure S4: Time series of PET material densities produced by CGenFF (blue) and Drude (red) models. Dashed gray line represents the experimental value obtained by Thompson and Woods[13].

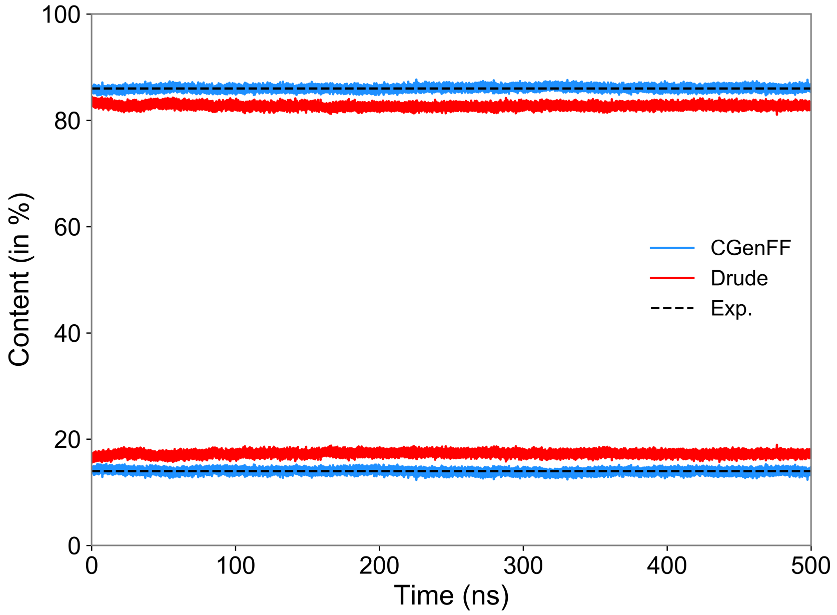

Figure S5: Time series of PET material *gauche* and *trans* populations sampled by CGenFF (blue) and Drude (red) models. Dashed gray lines represents the experimental values obtained by Schmidt-Rohr et al.[14].

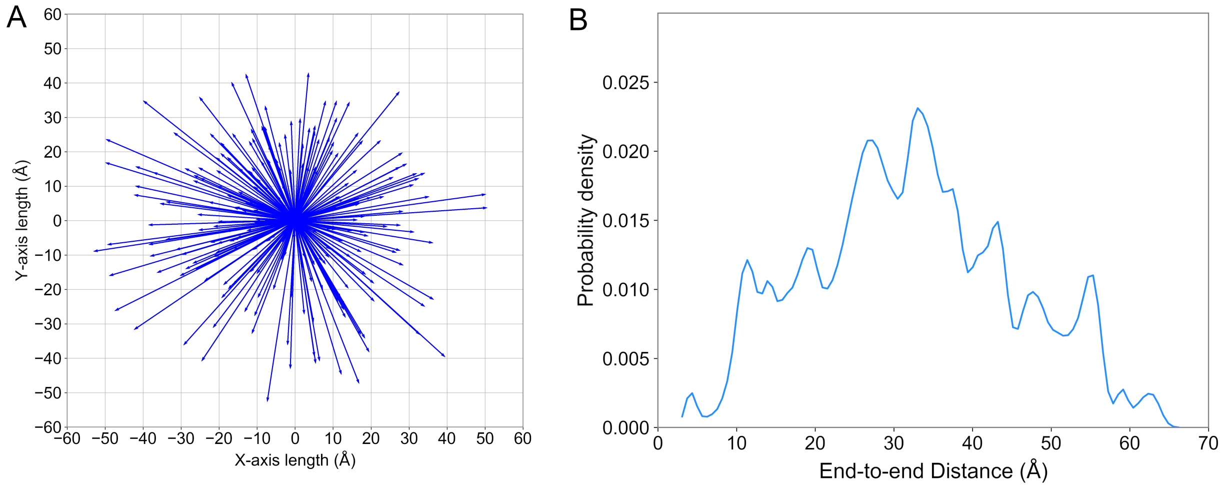

Figure S6: End-to-end distance vectors of the amorphous PET system. A) End-to-end vectors projected onto the xy-plane highlighting the random orientations of the PET chains. For comparison purposes, all vector origins were translated to (0,0,0). B) Distribution of the end-to-end distances of PET chains.

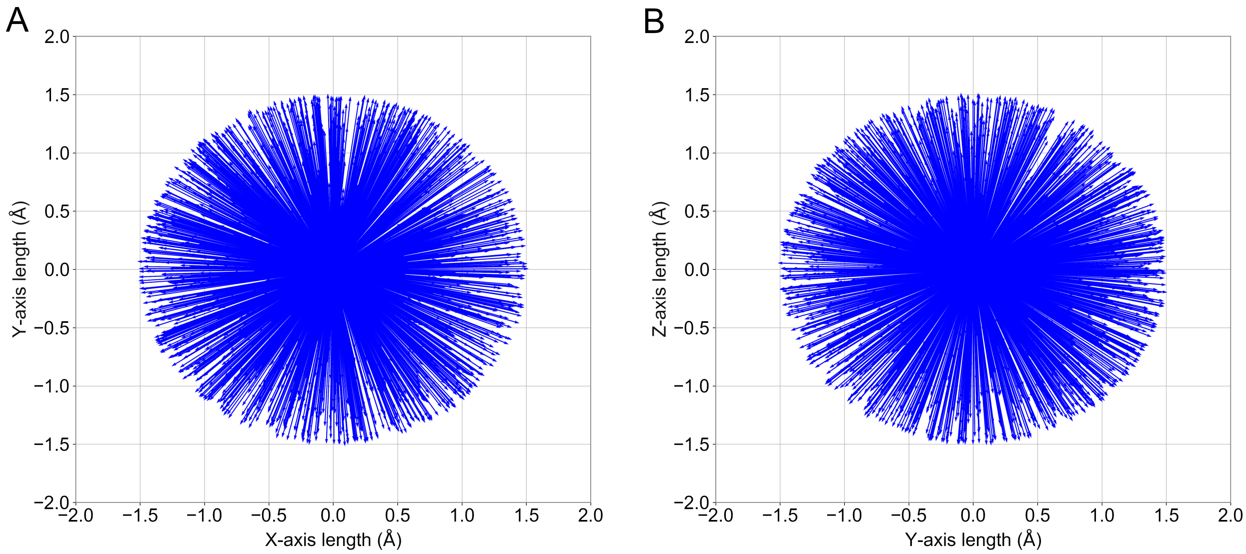

Figure S7: Bond vectors of PET chains in the amorphous system. A) PET linkage bond vectors projected onto the xy-plane highlighting the random organization of PET chains in the system. B) Same bond vectors now projected onto the yz-plane. For comparison purposes, all vector origins were translated to (0,0,0).

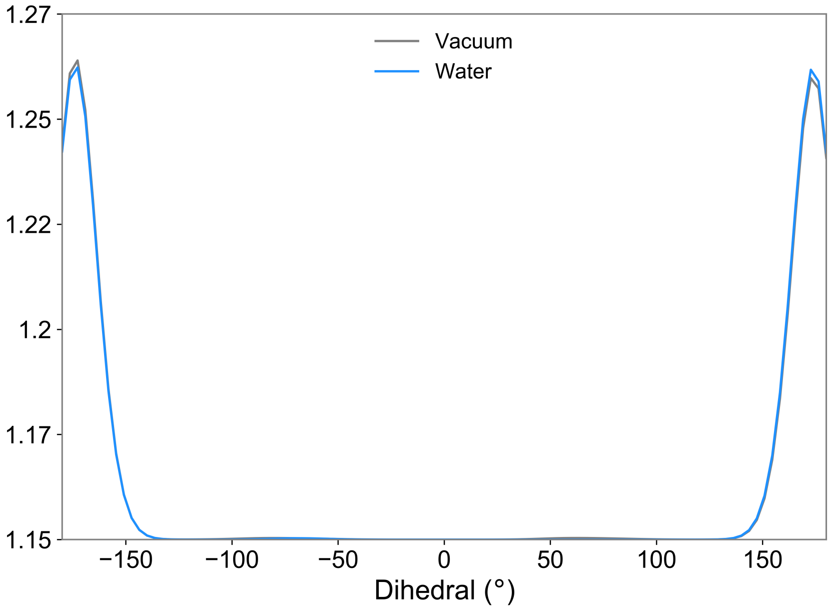

Figure S8: Distribution of cumulative Ψ values sampled by crystalline PET residues modeled by additive and Drude parameters in water.

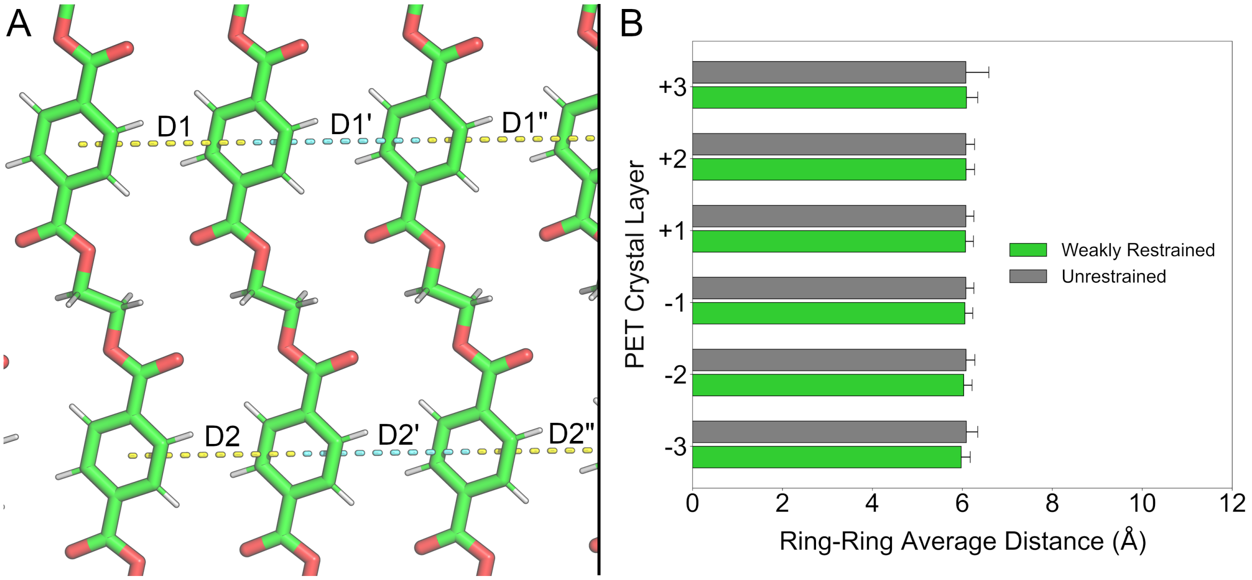

Figure S9: Average ring spacing in PET crystal unit in solution. A) Scheme used to calculate each ring-to-ring distance in a PET crystal layer. Distances were averaged for each crystal layer. B) Average ring-to-ring distance in each crystal layer for the system with a weak restraining potential applied to the bottom layer (green) and for the unrestrained system (gray).

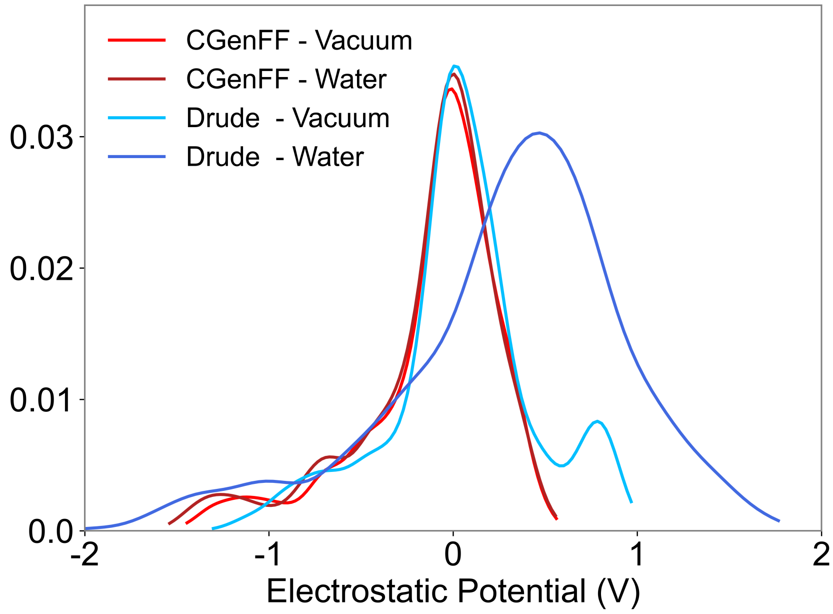

Figure S10: Distribution of electrostatic potential (in Volts) values at PET crystal lattice surface simulated in vacuum and in water and modeled by additive and Drude parameters.

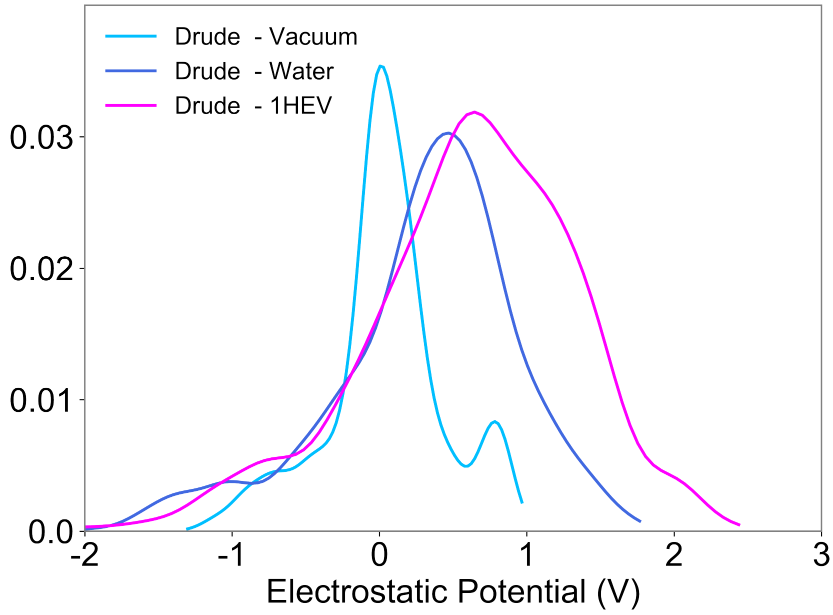

Figure S11: Distribution of electrostatic potential (in Volts) values at PET crystal lattice surface for Drude systems, showing an increase in potential values (in comparison to systems in vacuum and in water) induced by the binding of the hevein protein at the crystal lattice surface.

References

(1) Frisch, M. J.; Trucks, G. W.; Schlegel, H. B.; Scuseria, G. E.; Robb, M. A.; Cheeseman, J. R.; Scalmani, G.; Barone, V.; Mennucci, B.; Petersson, G. A.; Nakatsuji, H.; Caricato, M.; Li, X.; Hratchian, H. P.; Izmaylov, A. F.; Bloino, J.; Zheng, G.; Sonnenberg, J. L.; Hada, M.; Ehara, M.; Toyota, K.; Fukuda, R.; Hasegawa, J.; Ishida, M.; Nakajima, T.; Honda, Y.; Kitao, O.; Nakai, H.; Vreven, T.; Montgomery, J. A., Jr.; Peralta, J. E.; Ogliaro, F.; Bearpark, M.; Heyd, J. J.; Brothers, E.; Kudin, K. N.; Staroverov, V. N.; Kobayashi, R.; Normand, J.; Raghavachari, K.; Rendell, A.; Burant, J. C.; Iyengar, S. S.; Tomasi, J.; Cossi, M.; Rega, N.; Millam, J. M.; Klene, M.; Knox, J. E.; Cross, J. B.; Bakken, V.; Adamo, C.; Jaramillo, J.; Gomperts, R.; Stratmann, R. E.; Yazyev, O.; Austin, A. J.; Cammi, R.; Pomelli, C.; Ochterski, J. W.; Martin, R. L.; Morokuma, K.; Zakrzewski, V. G.; Voth, G. A.; Salvador, P.; Dannenberg, J. J.; Dapprich, S.; Daniels, A. D.; Farkas, Ö.; Foresman, J. B.; Ortiz, J. V.; Cioslowski, J.; Fox, D. J. Gaussian 09 Revision E.01.

(2) Brooks, B.; Brooks, C.; MacKerell, A.; Nilsson, L.; Petrella, R.; Roux, B.; Won, Y.; Archontis, G.; Bartels, C.; Boresch, S.; Caflisch, A.; Caves, L.; Cui, Q.; Dinner, A.; Feig, M.; Fischer, S.; Gao, J.; Hodoscek, M.; Im, W.; Kuczera, K.; Lazaridis, T.; Ma, J.; Ovchinnikov, V.; Paci, E.; Pastor, R.; Post, C.; Pu, J.; Schaefer, M.; Tidor, B.; Venable, R. M.; Woodcock, H. L.; Wu, X.; Yang, W.; York, D.; Karplus, M. CHARMM: The Biomolecular Simulation Program. *Journal of computational chemistry* **2009**, *30*, 1545–1614.

(3) Smith, D. G. A.; Burns, L. A.; Simmonett, A. C.; Parrish, R. M.; Schieber, M. C.; Galvelis, R.; Kraus, P.; Kruse, H.; Di Remigio, R.; Alenaizan, A.; James, A. M.; Lehtola, S.; Misiewicz, J. P.; Scheurer, M.; Shaw, R. A.; Schriber, J. B.; Xie, Y.; Glick, Z. L.; Sirianni, D. A.; O'Brien, J. S.; Waldrop, J. M.; Kumar, A.; Hohenstein, E. G.; Pritchard, B. P.; Brooks, B. R.; Schaefer, H. F., III; Sokolov, A. Y.;

Patkowski, K.; DePrince, A. E., III; Bozkaya, U.; King, R. A.; Evangelista, F. A.; Turney, J. M.; Crawford, T. D.; Sherrill, C. D. PSI4 1.4: Open-source software for high-throughput quantum chemistry. *The Journal of Chemical Physics* **2020**, *152*, 184108.

(4) Kirkpatrick, S.; Gelatt, C. D.; Vecchi, M. P. Optimization by Simulated Annealing. *Science* **1983**, *220*, 671–680, Publisher: American Association for the Advancement of Science.

(5) Lemkul, J. A.; Huang, J.; Roux, B.; Mackerell, A. D. An Empirical Polarizable Force Field Based on the Classical Drude Oscillator Model: Development History and Recent Applications. *Chemical Reviews* **2016**, *116*, 4983–5013, Publisher: American Chemical Society.

(6) Lin, F. Y.; Huang, J.; Pandey, P.; Rupakheti, C.; Li, J.; Roux, B.; Mackerell, A. D. Further Optimization and Validation of the Classical Drude Polarizable Protein Force Field. *Journal of Chemical Theory and Computation* **2020**, *16*, 3221–3239, Publisher: American Chemical Society.

(7) Kognole, A. A.; Aytenfisu, A. H.; MacKerell, A. D. Balanced polarizable Drude force field parameters for molecular anions: phosphates, sulfates, sulfamates, and oxides. *Journal of Molecular Modeling* **2020**, *26*, 152.

(8) Lamoureux, G.; Harder, E.; Vorobyov, I. V.; Roux, B.; MacKerell, A. D. A polarizable model of water for molecular dynamics simulations of biomolecules. *Chemical Physics Letters* **2006**, *418*, 245–249.

(9) Boys, S.; Bernardi, F. The calculation of small molecular interactions by the differences of separate total energies. Some procedures with reduced errors. *Molecular Physics* **1970**, *19*, 553–566, Publisher: Taylor & Francis _eprint: https://doi.org/10.1080/00268977000101561.

(10) Ransil, B. J. Studies in Molecular Structure. IV. Potential Curve for the Interaction of Two Helium Atoms in Single-Configuration LCAO MO SCF Approximation. *The Journal of Chemical Physics* **1961**, *34*, 2109–2118.

(11) Harder, E.; Anisimov, V. M.; Vorobyov, I. V.; Lopes, P. E. M.; Noskov, S. Y.; MacKerell, A. D.; Roux, B. Atomic Level Anisotropy in the Electrostatic Modeling of Lone Pairs for a Polarizable Force Field Based on the Classical Drude Oscillator. *Journal of Chemical Theory and Computation* **2006**, *2*, 1587–1597, Publisher: American Chemical Society.

(12) Tse, J. S.; Mak, T. C. W. Refinement of the crystal structure of polyethylene terephthalate. *Journal of Crystal and Molecular Structure* **1975**, *5*, 75–80.

(13) Thompson, A. B.; Woods, D. W. Density of Amorphous Polyethylene Terephthalate. *Nature* **1955**, *176*, 78–79, Publisher: Nature Publishing Group.

(14) Schmidt-Rohr, K.; Hu, W.; Zumbulyadis, N. Elucidation of the Chain Conformation in a Glassy Polyester, PET, by Two-Dimensional NMR. *Science* **1998**, *280*, 714–717, Publisher: American Association for the Advancement of Science.
